# Development of PF-06671008, a Highly Potent Anti-P-cadherin/Anti-CD3 Bispecific DART Molecule with Extended Half-Life for the Treatment of Cancer

**DOI:** 10.3390/antib5010006

**Published:** 2016-03-04

**Authors:** Adam R. Root, Wei Cao, Bilian Li, Peter LaPan, Caryl Meade, Jocelyn Sanford, Macy Jin, Cliona O’Sullivan, Emma Cummins, Matthew Lambert, Alfredo D. Sheehan, Weijun Ma, Scott Gatto, Kelvin Kerns, Khetemenee Lam, Aaron M. D’Antona, Lily Zhu, William A. Brady, Susan Benard, Amy King, Tao He, Lisa Racie, Maya Arai, Dianah Barrett, Wayne Stochaj, Edward R. LaVallie, James R. Apgar, Kristine Svenson, Lidia Mosyak, Yinhua Yang, Gurunadh R. Chichili, Liqin Liu, Hua Li, Steve Burke, Syd Johnson, Ralph Alderson, William J. J. Finlay, Laura Lin, Stéphane Olland, William Somers, Ezio Bonvini, Hans-Peter Gerber, Chad May, Paul A. Moore, Lioudmila Tchistiakova, Laird Bloom

**Affiliations:** 1 Global Biotherapeutics Technologies, Pfizer Inc., 610 Main St., Cambridge, MA 02139, USA; wei.cao@pfizer.com (W.C.); bilian.li@pfizer.com (B.L.); peter.lapan@pfizer.com (P.L.); caryl.meade@pfizer.com (C.M.); jocelyn.sanford@gmail.com (J.S.); macy.jin@pfizer.com (M.J.); weijun.ma@pfizer.com (W.M.); scott.gatto@pfizer.com (S.G.); kelvin.kerns@pfizer.com (K.K.); nee.lam@pfizer.com (K.L.); aaron.dantona@pfizer.com (A.M.D.); lily.zhu@pfizer.com (L.Z.); wmabrady@gmail.com (W.A.B.); susan.benard@pfizer.com (S.B.); amy.king@pfizer.com (A.K.); tao.he@pfizer.com (T.H.); lisa.racie@pfizer.com (L.R.); maya.arai@pfizer.com (M.A.); dianah.barrett@pfizer.com (D.B.); wayne.stochaj@pfizer.com (W.S.); edward.lavallie@pfizer.com (E.R.L.); james.r.apgar@pfizer.com (J.R.A.); kristine.svenson@pfizer.com (K.S.); lidia.mosyak@pfizer.com (L.M.); laura.lin@pfizer.com (L.L.); stephane.olland@gmail.com (S.O.); will.somers@pfizer.com (W.S.); lioudmila.tchistiakova@pfizer.com (L.T.); laird.bloom@pfizer.com (L.B.); 2 Global Biotherapeutics Technologies, Pfizer Inc., Grange Castle Business Park, Clondalkin, Dublin 22, Ireland; cliona_2000@yahoo.ie (C.O.); emmacummins@hotmail.com (E.C.); matthew.lambert@pfizer.com (M.L.); alfredo.sheehan@pfizer.com (A.D.S.); jonny.finlay@gmail.com (W.J.J.F.); 3 MacroGenics Inc., 9640 Medical Center Drive, Rockville, MD 20850, USA; yangy@Macrogenics.com (Y.Y.); chichilig@Macrogenics.com (G.R.C.); liul@Macrogenics.com (L.L.); lih@Macrogenics.com (H.L.); burkes@Macrogenics.com (S.B.); johnsons@Macrogenics.com (S.J.); aldersonr@Macrogenics.com (R.A.); bonvinie@Macrogenics.com (E.B.); moorep@Macrogenics.com (P.A.M.); 4 Oncology Research Unit, Pfizer Inc., 401 N. Middletown Road, Pearl River, NY 10965, USA; hanspeter.geber@pfizer.com (H.-P.G.); chad.may@pfizer.com (C.M.)

**Keywords:** P-cadherin, bispecific, cancer, immuno-oncology, T-cell, re-targeting

## Abstract

Bispecific antibodies offer a promising approach for the treatment of cancer but can be challenging to engineer and manufacture. Here we report the development of PF-06671008, an extended-half-life dual-affinity re-targeting (DART^®^) bispecific molecule against P-cadherin and CD3 that demonstrates antibody-like properties. Using phage display, we identified anti-P-cadherin single chain Fv (scFv) that were subsequently affinity-optimized to picomolar affinity using stringent phage selection strategies, resulting in low picomolar potency in cytotoxic T lymphocyte (CTL) killing assays in the DART format. The crystal structure of this disulfide-constrained diabody shows that it forms a novel compact structure with the two antigen binding sites separated from each other by approximately 30 Å and facing approximately 90° apart. We show here that introduction of the human Fc domain in PF-06671008 has produced a molecule with an extended half-life (~4.4 days in human FcRn knock-in mice), high stability (T_m_1 > 68 °C), high expression (>1 g/L), and robust purification properties (highly pure heterodimer), all with minimal impact on potency. Finally, we demonstrate *in vivo* anti-tumor efficacy in a human colorectal/human peripheral blood mononuclear cell (PBMC) co-mix xenograft mouse model. These results suggest PF-06671008 is a promising new bispecific for the treatment of patients with solid tumors expressing P-cadherin.

## 1. Introduction

Classical cadherins (E-, N-, and P-cadherin) comprise a family of molecules that mediate calcium-dependent cell-cell adhesion and are localized at the adherens junctions. P-cadherin overexpression has been reported to correlate with increased tumor cell motility and invasiveness [1,2,3,4]. Upregulation of P-cadherin has been reported in various tumors, including breast, gastric, endometrial, colorectal and pancreatic cancers, and is correlated with poor survival of breast cancer patients [4,5,6,7,8]. In contrast, low levels of the P-cadherin gene expression have been detected in normal tissues [6]. P-cadherin therefore represents an interesting target for the treatment of solid tumors that show increased levels of expression.

Bispecific antibodies (bsAb) have emerged in recent years as promising agents for immune-mediated tumor cell killing. The approval by the US Food and Drug Administration (FDA) of the bispecific T cell engager blinatumomab [9] for the treatment of relapsed or refractory B cell precursor acute lymphoblastic leukemia, as well as preclinical studies with additional bispecific platforms including the dual-affinity re-targeting (DART) scaffold [10], have demonstrated the potential role for bsAbs as potent cancer immunotherapies. BsAbs may also offer an opportunity for targeting cell-surface antigens that are not suitable for other therapeutic approaches such as antibody drug conjugates (ADCs, which normally require efficient internalization) or antibody-dependent cellular cytotoxicity (which may require high target expression levels) [11,12]. T cells express T cell receptor (TCR) complexes that are able to induce antigen-specific immune responses [13]. Engagement of antigen peptide/major histocompatibility complex (MHC) Class I on the target cell with the TCR induces the formation of an immune synapse and leads to signaling through CD3 co-receptors, which are components of the TCR signaling complex. This signaling cascade directs T cell-mediated killing of the cell expressing the antigen through the release and transfer of granzymes and perforin from the T cell to the target cell (reviewed in Voskoboinik *et al.*, 2015 [14]). In cancer, bsAbs that co-engage the CD3 epsilon subunit and a surface antigen on the tumor cell trigger T cell-mediated killing of the tumor cell while circumventing the need for the direct interaction of the TCR and MHC class I in complex with antigen. This expands the repertoire of T cells able to recognize the tumor and act as effector cells [15].

While bsAbs show great potential in opening up new therapeutic targets, many challenges remain, including difficulty in manufacturing, poor stability and short half-life (reviewed in Lameris *et al.*, 2014 [10]). Here we report the development of PF-06671008, a potent, stable, and manufacturable DART bsAb with extended *in vivo* half-life.

## 2. Results

### 2.1. Identification and Generation of Anti-P-Cadherin/Anti-CD3 DART Proteins

**Figure 1:**
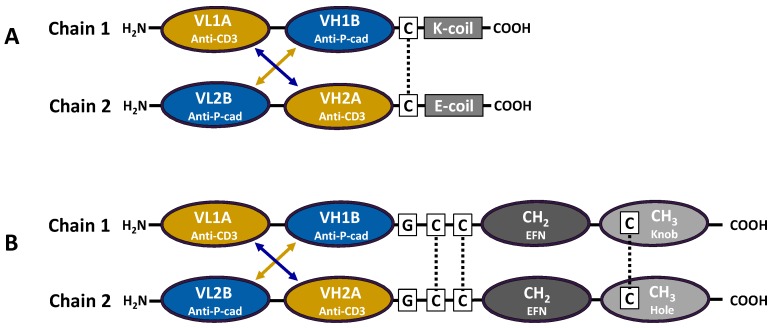
(**A**) Schematic representation of an anti-P-cadherin dual-affinity re-targeting (DART) construct containing E (glu) and K (lys) coiled-coil heterodimerization domains. Cysteine residues introduced for interchain disulfide formation are indicated by C; (**B**) Schematic representation of the anti-P-cadherin human IgG1 Fc-containing DART construct, also referred to as LP-DART. Effector function null (EFN) mutations in CH2; Knob, hole: complementary mutations introduced to force Fc heterodimerization [18,19].

Single chain variable fragments (scFv) with specific binding activity to P-cadherin were discovered through phage display followed by screening against recombinant soluble P-cadherin and cell surface-expressed P-cadherin in a direct binding enzyme-linked immunosorbent assay (ELISA) (Figure S1A,B). Clones demonstrating strong binding activity to both recombinant protein (optical density (OD)450 nm > 1) and cells expressing P-cadherin (OD450 nm > 0.8) were selected. These scFv were cloned into the DART format (Figure 1A) as previously described [16,17]. Oppositely charged coiled-coil sequences (E/K coils) were added at the carboxy terminus of each chain to drive heterodimerization. The introduction of a disulfide bond through added cysteines between the variable domains and the E/K coils on each chain further stabilizes this structure. Following transient expression in HEK293 cells, conditioned medium was screened in a bispecific ELISA format that uses CD3 for capture and either human or cynomolgus monkey P-cadherin extracellular domain (ECD) for detection (Figure S2), and active molecules were subsequently purified. DART proteins demonstrated high purity and stability (T_m_1 > 67 °C) by analytical size exclusion chromatography (SEC) and differential scanning calorimetry (DSC), respectively (Figure S3C,D). Non-reducing LC/MS analysis and SDS-PAGE exhibited pure polypeptide chains at the expected molecular weight (MW) of 57 kDa (Figure S3A,B).

### 2.2. Binding Properties of Anti-P-Cadherin DART Proteins to CD3 and P-Cadherin

**Figure 2:**
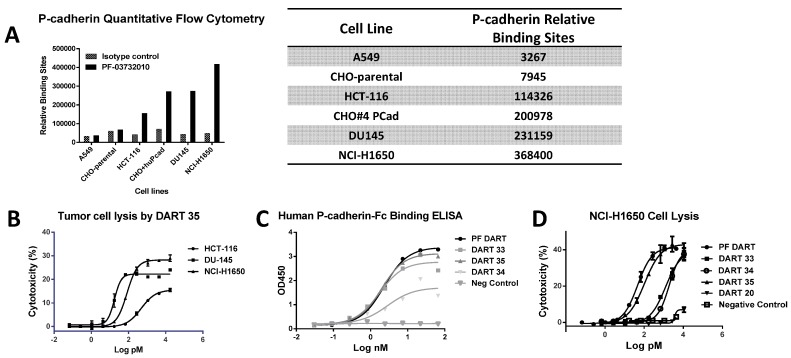
(**A**) Quantitative flow cytometry using phycoerythrin (PE)-conjugated anti-P-cadherin [20] at a fluorophore-to-protein ratio of 1:1. Relative antibody binding sites correlating to the number of antigens on the cell surface were determined using Quantum Simply Cellular Kit (Bangs Laboratories); (**B**) Graphical Cytotoxic T Lymphocyte (CTL) assay results of DART 35 on HCT-116, DU145 and NCI-H1650 cells; Graphical representation of (**C**) protein binding enzyme-linked immunosorbent assay (ELISA) and (**D**) NCI-H1650 adenocarcinoma CTL Assay.

To determine P-cadherin expression levels, several cell lines were analyzed by quantitative flow cytometry (QFACS) with an anti-P-cadherin monoclonal antibody PF-03732010 [20] labeled 1:1 with phycoerythrin (PE; Figure 2A). Expression levels were determined for the tumor cell lines naturally expressing P-cadherin including HCT-116 (colorectal; an antibody-binding capacity (ABC) of 114,000), NCI-H1650 (adenocarcinoma; ABC 368,000), DU-145 (prostate; ABC 231,000), or Chinese hamster ovary (CHO) cells engineered to express P-cadherin (ABC 201,000). Binding of PE-labeled PF-03732010 to A549 (adenocarcinoma) cells was similar to its binding to untransfected CHO cells and was also similar to binding of the isotype control to both cell types, indicating little or no P-cadherin expression, as previously described (Figure 2A) [21].

Specific binding activity of the anti-P-cadherin/anti-CD3 DART proteins was determined using dose-dependent binding ELISAs and/or surface plasmon resonance (SPR) assays run on a Biacore instrument (Figure 2C, Figures S4 and S5 and Table 1, Table S1). A panel of anti-P-cadherin/anti-CD3 DART proteins numbered 22, 30, 33, 34, 35; a positive control DART molecule (PF DART); and a negative control DART molecule (4420) were tested for binding against soluble human, cynomolgus monkey, and murine P-cadherin and against soluble human CD3epsilon-delta (εδ). All DART proteins tested bound to human and cynomolgus monkey P-cadherin with equilibrium dissociation (K_D_) constants in the range of 1–130 nM. DARTs 33, 34 and 35 appeared to recognize a different epitope than DARTs 20, 30 and PF based on the observation that 33, 34 and 35 did not recognize murine P-cadherin while the other DART molecules did (Figure S4). To determine binding to cell surface-expressed P-cadherin, tumor lines were used for cell binding ELISAs. These DART proteins bound to NCI-H1650 adenocarcinoma cells with effective concentrations at 50% (EC_50_) values between 20 and 142 nM while showing no binding to CHO cells that do not express P-cadherin (Table 1). P-cadherin shares moderate homology in the ECD with other classical cadherins [22]. However, no binding of the anti-P-cadherin/anti-CD3 DART proteins to either E-cadherin or VE-cadherin (46% and 27% amino acid identity, respectively, to P-cadherin ECD) was detected (Table S1C). Binding activity against soluble human CD3 epsilon was also tested. SPR equilibrium constants were observed in the range of 5–24 nM (Table 1).

### 2.3. Cytotoxic T Lymphocyte-(CTL) Directed Lysis of P-Cadherin-Expressing Tumor Lines by Anti-P-Cadherin/Anti-CD3 DART Proteins

To assess the *in vitro* potency of effector cell-redirected lysis by this same panel of anti-P-cadherin/anti-CD3 DART molecules, P-cadherin-expressing tumor cells were incubated with increasing concentrations of DART proteins and with either a 10 or 30-fold excess of peripheral blood mononuclear cells (PBMC) or purified T cells as effector cells. Lysis was measured after 24 or 48 h. Engineered CHO cells expressing P-cadherin demonstrated a similar level of P-cadherin expression as DU145 cells as determined by QFACS (Figure 2A). The maximum extent of cell killing of DART 35 in CTL-directed lysis of these cell lines correlated with P-cadherin expression level (Figure 2B). However, the potency did not correlate well with expression level. While all DART proteins showed specific binding to cell surface-expressed P-cadherin, only DARTs 33, 34 and 35 showed P-cadherin-specific lysis, with EC_50_ values in the range of 0.1–4 nM on tumor lines and 0.03–0.74 nM on CHO cells transfected with P-cadherin (Figure 2A, Figure S5B, and Table 1). DART 20 showed negligible, if any tumor cell lysis while DART 30 did not exhibit CTL activity. The positive control demonstrated the most potent CTL activity with EC_50_ values of 0.046–0.06 nM against tumor cells and 0.028 nM against CHO-P-cadherin cells (Table 1 and Figure S5A,B).

### 2.4. Selection of Anti-P-Cadherin Antibody Clone 153 from Affinity Optimization Screening

In an effort to increase the potency of DART proteins through improved binding affinity to P-cadherin, two strategies were attempted. First, a library of DART 35 variants was built through error-prone PCR of the anti-P-cadherin variable heavy (VH) domain randomly recombined with a library of naïve variable light (VL) domains. The DART 35 construct was selected for both its good CTL activity as well as its high germline sequence content (Table 2). This mutagenized library was then used for phage selections against both soluble protein and cells expressing P-cadherin, where the focus was on selecting for improved off-rates. Using a selection method termed “Hammer-Hug” [23], a highly-stringent first round was performed with a low concentration of biotinylated target antigen and overnight off-rate competition using non-biotinylated target, followed by a rescue round with increased target antigen concentration and no off-rate challenge. Additionally, cell based phage selections were performed using CHO-human P-cadherin engineered cells, including a 2 h off-rate competition with 200 nM human P-cadherin Fc.

A parallel affinity optimization strategy was used for the DART 34 molecule, which is highly similar to DART 35 in sequence and potency. A soft randomization approach was used to target each complementarity determining region (CDR) loop of the DART 34 anti-P-cadherin binding domain, with the exception of VL CDR2. Separate spiking mutagenesis libraries were built with mutagenic primers designed to encode approximately 50% wild-type and 50% any other amino acid at each position across each CDR [23]. Libraries were pooled, and phage selections were performed on biotinylated human P-cadherin-Fc for three rounds with decreasing target concentration, ending at 0.1–0.5 nM.

**Figure 3:**
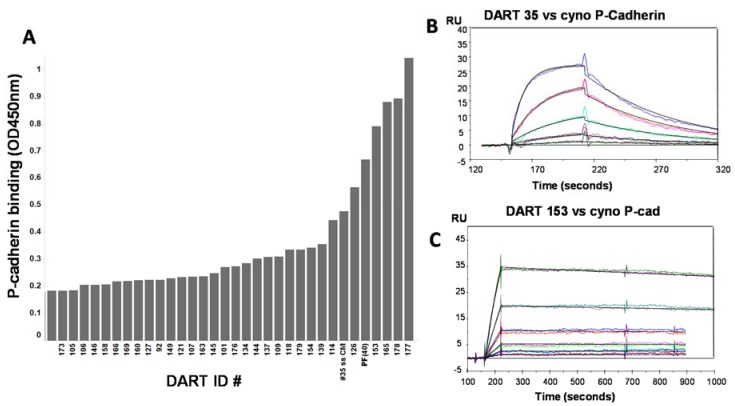
(**A**) Direct P-cadherin binding ELISA data (OD450 nm) of conditioned media samples of affinity matured DARTs compared to parental DART 35 and prototype (PF) DART. Surface plasmon resonance (SPR) Biacore sensogram shows (**B**) DART 35 and (**C**) DART 153 binding to cynomolgus P-cadherin extracellular domain (ECD) immobilized on the surface. Colored lines represent the fit to a 1:1 Langmuir model of the experimental binding curves obtained at DART concentrations of 1.6, 3.1, 6.3, 12.5, 25 or 50 nM.

Individual colonies infected with phage were picked from outputs of both optimization strategies and screened for improved binding properties by protein and cell ELISAs. Clones exhibiting improved binding compared to parental were subsequently reformatted into DART proteins for characterization in conditioned medium. Affinity-optimized DART proteins bound more strongly to human P-cadherin than the parental DART 35 in ELISA, and several clones also demonstrated stronger binding than the positive control (Figure 3A). A panel of DART molecules showing improved binding properties was selected for purification. Optimized DART constructs exhibited similar biophysical characteristics to those of parental DART 35, including DSC melting profiles (Figure S6). Binding affinity of optimized DART proteins to human CD3 measured by SPR was essentially unchanged from that of the parental clone. However, binding to cynomolgus monkey P-cadherin improved between 10- and 180-fold compared to DART 35. DARTs 165 and 177 showed K_D_s of 370 and 483 pM respectively (Table 3 and Table S2A,B). DART 153, which was selected from the cell/off-rate branch of the phage affinity-optimization selection, demonstrated a binding affinity of 231 pM, an increase of 187.5-fold compared to DART 35. This large increase in affinity was largely attributed to an 800-fold decrease in the dissociation rate (k_d_) (Table S2). Accurate K_D_ values for binding to human P-cadherin could not be determined due to antigen heterogeneity, but large gains in potency in CTL assays on cells expressing human P-cadherin were observed (see below).

Amino acid sequence alignment of the VH and VL CDRs of parental clones 34 and 35 and the affinity-optimized clones shows a small number of changes, primarily in CDR-H3. One clear trend among the high affinity binders is the substitution of an asparagine for a serine in CDR-H3 at position 99 (Table 3). Interestingly, asparagine at position 99 was also a common feature of optimized clones arising from more extensive randomization of CDR-H3 (Table 3). In addition, although the DART 35 optimization library contained a diverse set of naïve human VL domains, all high-affinity clones contained nearly the same framework and CDR sequences as the parental clone, with small variations in CDR-L3 (Table 3).

### 2.5. Cytotoxic T-Lymphocyte (CTL) Directed Lysis of P-Cadherin-Expressing Tumor Lines By Affinity-Optimized Anti-P-Cadherin/Anti-CD3 DART Proteins

To assess the improvement of *in vitro* CTL potency of the affinity matured anti-P-cadherin DART proteins, the same panel of tumor cell lines was tested as described above. CTL-directed cytotoxicity data shown in Table 3 and Figure S7A–C suggest that improved affinity correlates with improved cytotoxicity. DART clones with the strongest binding affinity such as 153, 165 and 177 also showed the strongest CTL activity, with EC_50_ values in the low to sub pM range. In the HCT-116 colorectal model, DART 153 showed an EC_50_ of 0.5 pM compared to 205 pM for parental DART 35, a 410-fold increase in potency accompanying a 187.5-fold increase in affinity.

### 2.6. Relationship between Binding Epitopes and T Cell-Retargeting Potency

**Figure 4:**
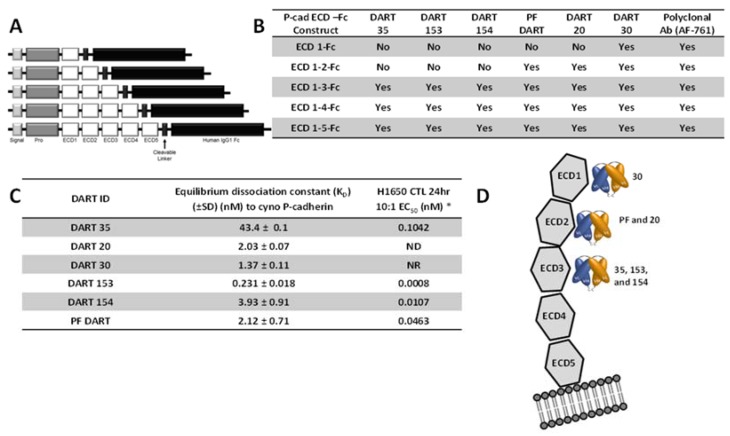
Epitope mapping of DARTs to the recombinant human P-cadherin extracellular domain. (**A**) Schematic representation of truncated human P-cadherin ECD constructs generated for epitope mapping studies. Gray shading represents the pro-peptide domain; White represents the P-cadherin ECD; Black shading represents the human IgG1 Fc with cleavable linker; (**B**) Summary of binding observed for P-cadherin DARTs to P-cadherin ECD-Fc constructs and the polyclonal anti-P-cadherin antibody (AF-761; R & D Systems); (**C**) CTL killing activity of DARTs tested in epitope mapping study; (**D**) Cartoon representation showing the presumed binding location of the various DARTs. * = samples were tested on a separate dates with different donor effector cells. ND = EC_50_ not determined; NR = no response.

To determine the epitope on P-cadherin extracellular domain (ECD) recognized by the anti-P-cadherin/anti-CD3 DART molecules, binding of DART proteins to a series of truncated human P-cadherin ECD-Fc fusions was tested (Figure 4, Figure S8). A polyclonal Ab (R & D Systems, Minneapolis, MN) confirmed proper expression of truncated extracellular domains. DART 35 and its optimized derivatives, 153 and 154, which are presumed to share the same epitope based on near-identical CDR sequences, show binding to all constructs that contain ECD3 but not to either ECD1-Fc or ECD1–2-Fc, suggesting that these DART proteins bind to human P-cadherin within ECD3. Similarly, DARTs 20 and PF bind to all constructs that contain ECD2 but not to ECD1-Fc alone, indicating a binding site within ECD2. DART 30 binds to a construct containing ECD-1 alone, indicating a binding site in this most distal domain. DART 35 does not bind to murine P-cadherin (Figure S4), so it is reasonable to postulate that based on binding analysis to the truncated human P-cadherin ECD-Fc constructs and to murine P-cadherin, DARTs 35, 153 and 154 recognize an epitope on ECD3 of human P-cadherin containing at least some of the 16 residues that differ in sequence between human and mouse P-cadherin ECD3.

Others have reported that epitope location in relation to the target cell membrane can affect efficiency and potency of redirected lysis [24]. A comparison of CTL assay potency of DARTs 30, 20, PF, and 154, which have affinities within three-fold of one another (Figure 4C), showed a trend toward higher potency corresponding to more membrane-proximal epitopes. Within a single epitope (DARTs 35, 153 and 154), increasing affinity corresponded with higher potency. DART 20 demonstrated strong binding to recombinant P-cadherin by ELISA and Biacore but weaker binding by cell ELISA (Table 1) and negligible, if any CTL activity (Figure 2D, Figure S5B). This raises the question as to whether DART 20 recognizes an epitope that is only poorly exposed on native P-cadherin.

### 2.7. Crystallography of P-Cadherin x CD3 DART 35 Molecule

**Figure 5:**
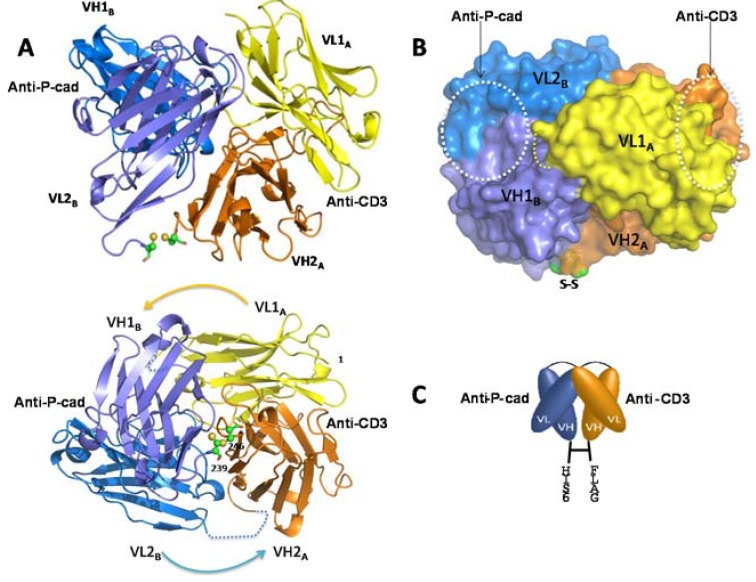
(**A**) Views from side (top) and above (bottom) of the crystal structure of DART 35 illustrate the tightly packed diabody formation. The structure shows that the disulfide bond (green) is formed between the two chains, keeping the diabody in a compact configuration; (**B**) Antigen binding sites for the anti-CD3 CDR region (right white dot circle) and the anti-P-cadherin CDR region (left white dot circle) of DART 35 are highlighted in the space-filled illustration. Yellow dot circle represents the interface region. The binding sites for the two antigens are located on opposite sides of the diabody; (**C**) Schematic representation of the DART 35 used for crystallography studies. Pcad = P-cadherin; VH = Heavy chain variable region; VL = Light chain variable region; S–S = Disulfide bond.

The spatial arrangement of the two binding domains in a bispecific molecule can have a major impact on its ability to crosslink its two binding partners. Crystallographic studies of the DART protein were undertaken using a version of DART 35 lacking the E and K coils, replaced instead with poly-histidine and FLAG tags for purification purposes (Figure 5C). This “coil-less” DART protein was transiently expressed and purified to high purity using anti-FLAG and anti-His chromatography (Figure S9A–C). The purified coil-less DART 35 demonstrated CTL-directed killing activity equivalent to that of the DART 35 construct containing the E and K coils (Figure S9D).

The crystal structure of the coil-less DART 35 protein was determined to 2.0 Å resolution (Figure 5). Crystallographic data collection, data processing and refinement statistics can be found in Table S3. The DART model shown in Figure 5 comprises two chains, H and L, with residues 1–110 and 117–239 of chain H and residues 2–110 and 116–246 of chain L present. Non-protein atoms present in the model include 574 water molecules and five sulfate ions. Missing amino acids in the linker regions were not modeled into the structure because of the lack of electron density, very likely due to disorder.

In the crystal, the DART 35 protein assembles into a tightly compact spherical structure that differs considerably from the previously published diabody structures (Figure 5A) [25,26]. Analysis of the interface area shows that the total buried surface area (BSA) between the two chains is 4532 Å^2^, which is significantly higher compared to the BSA of ~3458 Å^2^ for the previously-published extended diabody structure (PDB accession No. 1MOE). The four subunits, VL1A, VH1B, VL2B, and VH2A, all contribute to the diabody interface, with the greatest contributions coming from the framework amino acid residues of both chains. The two antigen binding sites are separated from each other by approximately 30 Å and are facing away from each other at an angle of ~90°. The anti-CD3 CDRs (right white circle in Figure 5B) are positioned approximately 22 Å away from the subunit interface (yellow circle in Figure 5B) whereas the anti-P-cadherin CDR regions (left white circle in Figure 5B) lie within a smaller 16 Å-radius area.

### 2.8. Fc-DART Protein Engineering for Extended Half-Life

The small size of bispecific antibody formats such as BiTEs and DARTs (~50 kD) can lead to fast clearance and a short half-life [17]. For improved pharmacokinetic properties, the anti-P-cadherin/anti-CD3 DART molecule was fused to a human IgG1 Fc domain, thereby increasing the molecular mass and incorporating the portions of the IgG1 molecule that achieve long half-life through interaction with FcRn [27]. We refer to this diabody-Fc fusion molecule as the LP-DART format (Figure 1B). To maximize yield of the desired heterodimeric diabody-Fc protein and to simplify purification, “knobs-into-holes” mutations were engineered into the Fc domain. With this design, Fc domains are driven to form heterodimers instead of their normal homodimers by addition of protruding bulky hydrophobic residues (“knobs”) to one chain and creation of complementary hydrophobic pockets (“holes”) on the other [18,19]. For added stability, a disulfide bond was introduced through the addition of a single cysteine in the CH3 domain of both the knob and hole chains. Furthermore, mutations were made in the lower hinge region to ameliorate unwanted antibody-dependent cell-mediated cytotoxicity (ADCC) [28].

Variants of LP-DART 35 (called LP-DARTs 1–8) with different linker lengths and alternate orientations of variable domains in relation to the knob and hole chains were expressed, purified and tested for activity and affinity (Table 4). While all Fc-bearing DART proteins showed a significant improvement in transient expression yield (20–35 mg/L up from 5 mg/L), aggregation (high molecular mass species or HMMS) was observed for all variants following Protein A capture, consistent with that which is typically seen for an Fc fusion protein. Biacore binding to P-cadherin and CD3 showed a modest (~1.5–3-fold) decrease in affinity to both targets and similar modest decreases in CTL activity (~2.2–8.6-fold). The construct with the highest retention of activity, LP-DART 5, had a single glycine residue in the linker connecting the *C*-terminus of the diabody to a truncated “core” hinge of the Fc domain, starting at the sequence CPPC (EU numbering 226–229). Several orientations of the VH and VL fragments were tested to determine optimal folding and expression profiles of the diabody when fused to an Fc domain. The optimal format consisted of anti-P-cadherin VL fused to anti-CD3 VH on the Knob Fc domain and anti-CD3 VL fused to anti-P-cadherin VH on the Hole Fc domain (Table 4).

### 2.9. Generation and Characterization of PF-06671008

DART 153, which combined the highest affinity, highest *in vitro* CTL potency, and fewest differences from human germline sequences, was cloned into the LP-DART format to generate PF-06671008. The two chains of PF-06671008 were cloned into a mammalian expression vector, pRY19-GA-Q, containing dual promoters and a recombination site for single-site integration into the CHO cell genome. Stably-transfected CHO cell pools were expanded and conditioned medium collected over a 12-day period. The LP-DART protein was purified utilizing an initial Protein A affinity capture step. The yield following Protein A elution from five separate harvests averaged 1.3 g/L.

**Figure 6:**
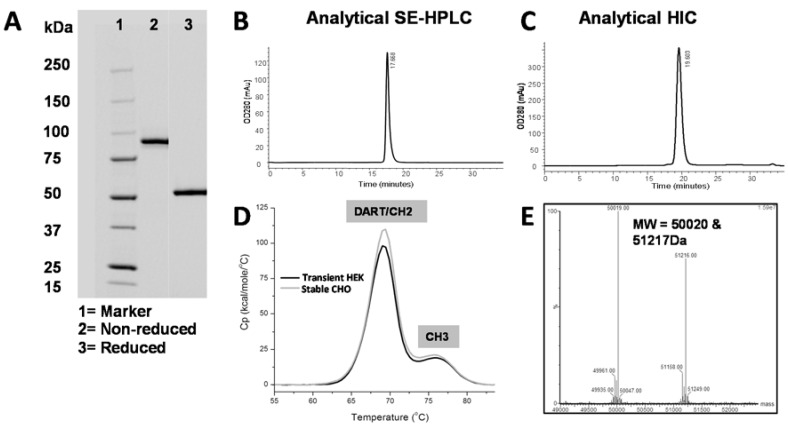
Biophysical properties of purified anti-P-cadherin LP-DART #153 (PF-06671008) (**A**) SDS-PAGE analysis under non-reducing and reducing conditions; (**B**) Size exclusion chromatography (SCE) of PF-06671008 on Superdex200 10/30GL column; (**C**) Analytical hydrophobic interaction chromatography (HIC) (**D**) Differential scanning calorimetry (DSC) analysis of transiently and stably expressed PF-06671008 exhibits thermal profiles with T_m_1 transitions ≥ 68 °C; (**E**) Liquid chromatography/ mass spectrometry (LC/MS) of PF-06671008: reduced/alkylated and PNGaseF treated PF-06671008 shows two peaks at 50019 and 51216, corresponding well with the predicted molecular weight (MW) of the two chains 50020 and 51217 respectively.

Following purification and formulation into PBS, PF-06671008 was characterized for biophysical properties (Figure 6A–E). Minimal HMMS was observed by analytical SEC, and minimal product-related impurity was observed by analytical hydrophobic interaction chromatography (HIC). DSC analysis showed a T_m_1 (diabody and CH2 domains) of >68 °C and a T_m_2 of >75 °C. SDS-PAGE and LC/MS analysis under reducing conditions confirm the predicted molecular weight of each chain of PF-06671008.

The binding properties of highly-purified PF-06671008 were assessed as before (Table 5). PF-06671008 bound to NCI-H1650 cells with an EC_50_ value of 0.593 nM, while no binding was detected to P-cadherin-negative CHO cells. SPR was used to confirm the affinity constants for PF-06671008 binding to cynomolgus monkey P-cadherin (0.521 nM) and human CD3 (11.5 nM). No binding was detected against the mouse P-cadherin by SPR, as expected. Cynomolgus monkey P-cadherin affinity data generated by KinExA compared well with binding data generated by Biacore. Binding to recombinant human P-cadherin measured by Biacore was strong but accurate KD values could not be determined, possibly due to heterogeneity of the commercially-available human P-cadherin protein. Affinity measurements by an orthogonal method using the KinExA instrument showed that PF-06671008 binding to cell surface-expressed human P-cadherin and to recombinant cynomolgus monkey P-cadherin protein had similar affinities, 176 and 352 pM, respectively.

### 2.10. Cytotoxic T-Lymphocyte (CTL) Directed Lysis of P-Cadherin-Expressing Tumor Lines by PF-06671008

**Figure 7:**
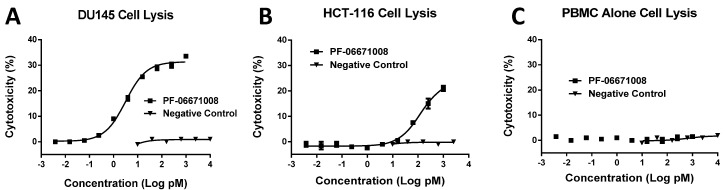
Dose-response curves of PF-06671008 or control DART (4420-LP)-mediated cytotoxicity measured using the lactate dehydrogenase (LDH) assay with primary human peripheral blood mononuclear cells (PBMCs) as effector cells and (**A**) DU145, or (**B**) HCT-116-Luc cell lines, respectively, as target cells at an effector:tumor (E:T) cell ratio of 30:1; (**C**) Dose-response curves of PF-06671008 or control DART (4420-LP)-mediated cytotoxicity measured using the LDH assay with primary human PBMCs alone.

The ability of PF-06671008 to support redirected T cell killing and cytokine release in the presence of human PBMCs was performed using two cancer cell lines, which were confirmed by flow cytometry (Figure 2A) to express a different levels of cell surface P-cadherin (DU145, 231,000 copies/cell, and HCT-116, 114,000 copies/cell). An effector:target (E:T) cell ratio of 30:1 was employed, and the level of target cell lysis was determined from the LDH release assay after 24 h of incubation. PF-06671008 mediated killing of the higher-expressing cells, DU145, with an EC_50_ of 3.1 pM, while it was less potent (122.6 pM EC_50_) on the lower-expressing line, HCT-116 (Figure 7, Table 6). The control DART (4420-LP), consisting of an irrelevant antibody against fluorescein coupled with the same anti-CD3 component as in PF-06671008, did not mediate redirected killing. Furthermore, in the presence of PBMCs alone, which are negative for P-cadherin expression, no PF-06671008-mediated LDH release was observed (see Figure 7C).

The ability of PF-06671008 to induce release of TNF-α, IFN-γ, IL-2, IL-4, IL-6, and IL-10 during incubation with human PBMCs alone or in the presence of target cells was also investigated. Cytokine levels were measured in parallel from supernatants collected from the LDH release assays following 24 h of incubation. Consistent with the absence of PF-06671008-mediated cytotoxicity observed with human PBMCs alone, no cytokine production was noted following PF-06671008 incubation with human PBMCs alone (Table 6B, Figure S10A). In contrast, dose-dependent PF-06671008-induced cytokine production was observed in the presence of target cells. The sensitivity to PF-06671008-mediated cytokine release and the maximal levels of cytokine release (E_max_) both tracked with the expression level of P-cadherin on target cells, with lower EC_50_ and higher E_max_ values observed with cells expressing higher levels of P-cadherin (Table 6B). Incubation with a control DART (4420-LP) did not result in detectable cytokine production (Figure S10).

### 2.11. Pharmacokinetic Properties of PF-06671008 in Human FcRn Knock-in Mice

**Figure 8:**
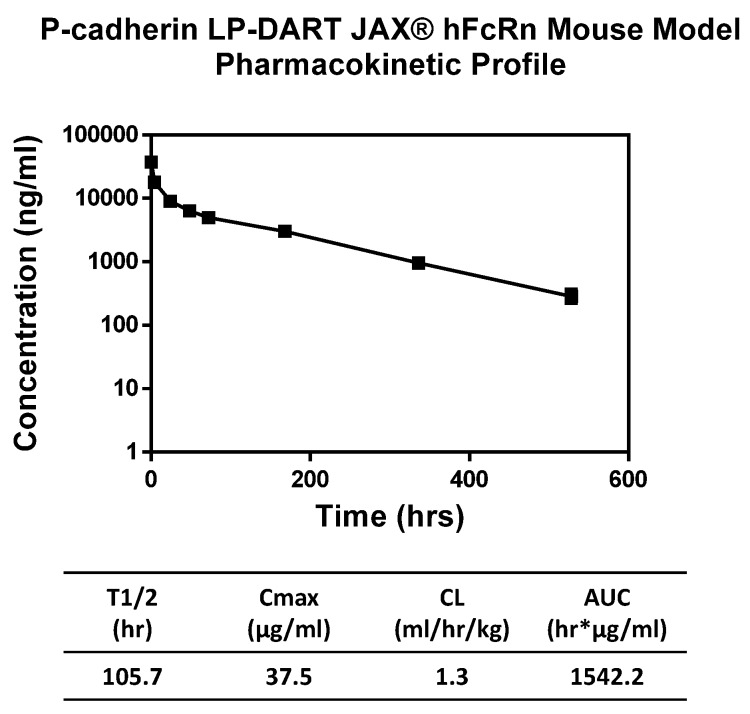
P-cadherin LP-DART serum concentrations following a single 2 mg/kg IV bolus are plotted (upper panel); the maximum observed concentration (C_max_), area under the concentration curve to the last quantifiable concentration (area under the curve or AUC), predicted total body clearance (CL), and terminal half-life (T_1/2_) determined by Non Compartment Analysis using WinNonlin are tabulated below. The DART molecule without the Fc domain was not tested in this study. However, previous studies of unrelated DART proteins in several strains of mice have shown rapid clearance, with T_1/2_ values ranging from 2.4 to 3.6 h [16].

Pharmacokinetic (PK) characteristics of PF-06671008 were determined after 2 mg/kg intravenous (IV) administration in human FcRn transgenic mice lacking normal mouse FcRn gene expression (Figure 8). Fully intact DART concentration was measured by ELISA at the following time points: pre, 5 min, 4 h, and day 1, 2, 3, 7, 14 and 22. Terminal half-life (T_1/2_) was determined to be 105.7 h or ~4.4 days (Figure 8). C_max_ was calculated to be 37.5 µg/mL with a clearance rate of 1.3 mL/h/kg and an area under the curve (AUC) of 1542.2 hr * µg/mL.

### 2.12. Inhibition of Tumor Growth by PF-06671008 in a Colorectal Xenograft Model

**Figure 9:**
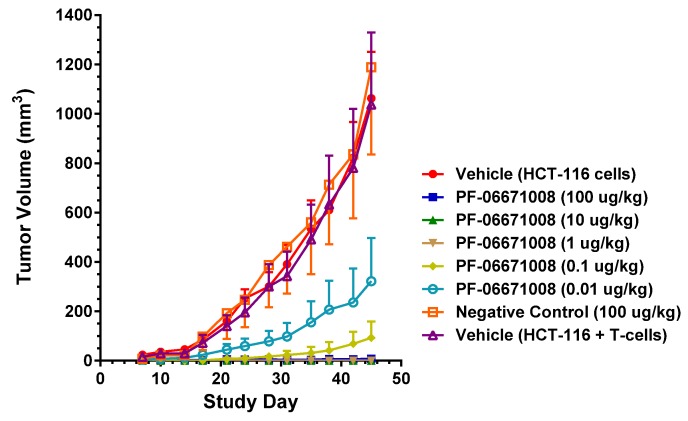
Inhibition of tumor growth by PF-06671008 in mice implanted with human colorectal tumor cells (HCT-116) in the presence of activated human T cells. Female NOD/SCID mice (*n* = 8/group) were implanted subcutaneously (SC) with HCT-116 tumor cells + human T cells on Day 0 followed by treatment with vehicle control, 100 µg/kg control DART (4420-hXR32-LP), or 0.01 to 100 µg/kg PF-06671008 on Days 0–3 for a total of four doses administered intravenously (IV). Tumor volume was measured through Day 45 (mean ± standard error of mean (SEM) is shown).

Activated human T cells (1 × 10^6^ cells) and HCT-116 human colorectal tumor cells (5 × 10^6^ cells) were premixed in a 1:5 effector to target ratio and implanted SC in female NOD/SCID mice on Day 0 (Table 7). Mice were then treated IV with vehicle control, 100 µg/kg control DART (4420-hXR32-LP), or PF-06671008 at five different dose levels (0.01, 0.1, 1, 10, 100 µg/kg) once daily for 4 days starting on the day of tumor cell implantation (Days 0, 1, 2, and 3). Figure 9 shows the effect over time of treatment with vehicle, control DART, or PF-06671008 on HCT-116 tumor growth in mice. Mice treated with vehicle or the control DART demonstrated HCT-116 tumor growth throughout the study. By Day 45, the time point at which the study was terminated, the tumors had reached mean (± SEM) volumes of 1063 ± 188 mm^3^, 1038 ± 291 mm^3^, and 1188 ± 353 mm^3^ in the vehicle (HCT-116 cells only), vehicle (T cells + HCT-116 cells), and control groups, respectively. For the 0.1 µg/kg (*p* < 0.05) and the 1 to 100 µg/kg (*p* < 0.01) PF-06671008 dose groups, significant inhibition of tumor growth compared with each of the three control groups was first observed on Day 35. For the 0.01 µg/kg dose group, significant tumor inhibition (*p* < 0.001) was first apparent on Day 42. At the end of the study (Day 45), inhibition of HCT-116 tumor growth remained statistically significant (*p* < 0.0001) following treatment with PF-06671008 at doses ≥ 0.01 µg/kg compared with the three control groups. In the lowest dose group (0.01 µg/kg), 4/8 mice had no detectable tumors (group mean = 322 ± 176 mm^3^), while in the 0.1 µg/kg dose group, 6/8 mice had no detectable tumors (group mean = 93 ± 66 mm^3^). Moreover, all animals appeared tumor-free with PF-06671008 doses ≥1 µg/kg.

## 3. Discussion

Here we describe the discovery and development of a potent anti-P-cadherin/anti-CD3 bispecific DART protein, PF-06671008, capable of redirecting CD3-positive effector cells to kill P-cadherin expressing cells. We provide an assessment of its preclinical *in vitro* and *in vivo* pharmacologic properties, as well as its manufacturability characteristics. We have engineered an extended half-life bispecific molecule that demonstrates antibody-like stability and that can be manufactured under conventional bioprocessing techniques without many of the challenges normally associated with bispecific antibodies.

P-cadherin has been extensively studied as a target for cancer therapy, and while its role may vary across different cancer types, increased P-cadherin expression is well documented in multiple tumor types and often correlates with poor survival. We showed that a bispecific antibody (bsAb) composed of variable regions from a P-cadherin antibody and variable regions from an anti-CD3 antibody had potent T cell-dependent cytotoxic activity on P-cadherin-expressing tumor cells. Using quantitative flow cytometry, we determined the expression level of several tumor lines and demonstrated that the potency of P-cadherin-dependent cell lysis tracked with P-cadherin expression. We observed potent cytotoxicity even with cells expressing relatively low copy number suggesting T cell retargeting through bsAbs offers a potential therapeutic modality to target tumors with moderate to low target expression levels.

A panel of specific anti-P-cadherin scFv was discovered through phage display selections with a naïve human library targeting recombinant P-cadherin protein and cell lines engineered to overexpress P-cadherin. These small antibody fragments, already expressed and functional in the scFv format, were easily re-cloned into the diabody-based DART format. Cell and protein binding studies of this panel of anti-P-cadherin/anti-CD3 DART proteins demonstrated specific reactivity to human and cynomolgus monkey P-cadherin but in some cases not murine P-cadherin.

T cell-directed lysis of tumor cells expressing P-cadherin by these novel anti-P-cadherin/anti-CD3 DART molecules showed moderate cell killing activity (EC_50_ ~0.3–3.6 nM) for some but not all DARTs tested. These data supported further optimization to determine if improved affinity could increase cytotoxic activity. Mutagenesis techniques designed to introduce small numbers of amino acid changes to CDRs of either of the closely-related anti-P-cadherin clones 34 or 35 were combined with stringent phage selection methods to yield a panel of affinity optimized clones. Changes of only one or two amino acids in the CDR regions led to significant improvements in equilibrium dissociation constants above the 43.4 nM affinity of the starting clone, sometimes over 100-fold (Table 3). For example, optimized DARTs 153 and 165 showed K_D_ values of 231 and 370 pM, respectively. For DART 153, a 187-fold improvement in binding affinity to P-cadherin translated to an increase of over 400-fold in CTL-directed potency in the HCT-116 colorectal tumor model, with an EC_50_ value of 0.5 pM. Improvement in affinity generally correlated well with improvement in CTL activity. DARTs showing only a modest improvement in affinity such as 154 (11-fold) and 179 (10-fold) showed the smallest improvement in CTL activity (~12-fold and 5-fold respectively). Sequence analysis of the optimized clones identified a serine to asparagine change at position 99 of the CDR-H3 as a common mutation that emerged from the selections and was present in clones from both phage optimization strategies. In addition, the phage selections revealed a strong preference for the parental light chain sequence: from a library in which the mutagenized VH chain of clone 35 was paired with a collection of random naïve human VL chains from an unselected phage library, all hit molecules retained the parental VL framework (VL1b.366F5/DPL5), with only minor CDR sequence variations. Since the affinity optimization was conducted with P-cadherin binding domains in scFv format, in the absence of the anti-CD3 domain, it is likely that these observed improvements reflect increases in binding to P-cadherin and not alterations to the DART structure. This is further supported by the fact that binding to CD3 remained largely the same following optimization.

In addition to affinity, the epitope on the P-cadherin extracellular domain had a major influence on the potency of effector cell directed-tumor cell lysis. A common structural configuration shared across classical, type II and desmosomal cadherins is a series of five extracellular domains termed ECD1 (membrane distal) to ECD5 (membrane proximal), separated by calcium-binding domains (reviewed by Shapiro and Weis, 2009 [29]). We used a set of truncated P-cadherin ECD constructs to show that DART 30 bound to ECD1, while DARTs 20 and PF bound to ECD2, and DART 35 and its optimized derivatives (153 and 154) bound more membrane-proximally, in ECD3. While DARTs 30, 20, PF, and 154 have similar binding affinity (1.4–3.9 nM), the cytotoxic potency is highest with the most membrane-proximal (DART 154, EC_50_ 0.01 nM), intermediate for the ECD 2-binding DARTs PF and 20 (0.4 and 34 nM, respectively), and absent for the most distal DART, 30. These results are consistent with those reported by Bluemel *et al.*, who showed that the distance of an epitope from the cell surface recognized by BiTE molecules to melanoma-associated chondroitin sulfate proteoglycan (MCSP), all with similar equilibrium dissociation constants, greatly influenced BiTE redirected lysis [24]. They postulate that epitopes closer to the cell surface allow for more efficient formation of the cytolytic synapse and delivery of perforins and granzymes [24].

Although the structure of P-cadherin is not known, the structure of C-cadherin has been solved to 3.1 Å [30]. This structure shows an elongated, slightly curved ectodomain stretching away from the cell surface, with an approximate total length of 193 Å (19.3 nm; [30], or ~3.9 nm for each domain. If P-cadherin has a similar structure, ECD3 would extend between approximately 11.6 and 15.4 nm from the cell surface, similar to the reported length of the TCR/peptide-MHC complex (~14 nm or 140 Å; [24]). Binding to ECD3 via a compact DART protein to the membrane-proximal CD3 molecule may provide the optimal distance for formation of the T cell-target cell synapse, while epitopes in more distal domains would reduce the likelihood of synapse formation. While these observations are based on the crystal structure of a recombinant cadherin-Fc fusion protein, *in vitro* CTL activity on tumor lines supports our hypothesis.

Here we report for the first time a crystal structure of a disulfide-constrained diabody. Diabodies can be engineered with V domains of different Fv regions mixed on opposite chains and can be separated by short linkers that prevent intra-chain VH-VL folding [31]. The 2 Å resolution crystal structure of coil-less DART 35, arranged in the VL-VH format and separated by a 9-residue intra-chain linker on chain 1 (CD3VL-P-cadVH) and an 8-residue linker on chain 2 (PcadVL-CD3VH), shows that this DART construct assembles into a tightly packed spherical structure that differs considerably from the previously published diabody structures (Figure 5A,B). The VH-VL diabody reported by Perisic *et al.* [25] (PDB accession No. 1LMK) consisted of short 5-residue intra-chain linkers and showed a symmetrical yet flexible structure. The anti-CEA diabody in VL-VH format, T84.66 (consisting of two identical scFv separated by longer, eight residue intra-chain linkers), takes on an asymmetrical shape as reported by Carmichael *et al.* [26] (PDB accession No. 1MOE).

The previously-published structures are elongated, with a wide 42 Å–60 Å gap separating the distal (non-CDR-containing) ends of the two Fv domains [25,26]. In contrast, the DART 35 structure reported here shows that the disulfide bond formed between the two chains keeps the diabody in a compact configuration and reduces the distance between the two domains to 5.3 Å. Addition of a *C*-terminal Gly-Gly-Cys to the T84.66 diabody allowed the formation of a stable, covalent disulfide-linked diabody [32,33], and Olafsen *et al.* proposed a structure in which the Fvs were rotated to bring the distal ends close enough for the disulfide bond formation, similar to the observed DART 35 structure. While the intra-chain linkers in the DART 35 are similar in size to those in T84.66, we did not observe these residues in our structure, likely due to disorder of the linkers.

The four subunits, VL1A, VH1B, VL2B, and VH2A shown in Figure 5A all participate in the diabody interface, with the most contribution coming from the framework amino acid residues of both chains. The centers of the two paratopes are separated from each other by approximately 30 Å and are facing ~90° apart. The orientation and short distance between the antigen binding domains of the DART protein may allow for more efficient T cell-target cell synapse formation and contribute to the increase in potency of T cell directed lysis observed with DART molecules compared to other bispecific antibody formats [17]. The anti-CD3 CDR region is positioned remotely from the subunit interface, whereas the anti-P-cadherin CDR region lies adjacent to the interface (Figure 5B). This observation suggests that swapping the order of the binding domains or introducing a different binding domain to either P-cadherin or CD3 may impact orientation and therefore bispecific binding activity. Key mutations in the anti-P-cadherin CDR which show significant change in affinity are at Kabat position H99 in the CDR-H3, and to a lesser extent Kabat positions L96 and L97 in CDR-L3 (Table 3). The H99 mutation to asparagine increases the affinity by between 10 and 187-fold depending on concomitant mutations and is predicted to either alter a potential surface interaction with P-cadherin or stabilize the loop conformation across the VH/VL interface of the P-cadherin-binding portion of the diabody. The L96 and L97 mutations which alter the affinity to a lesser extent are at the base of the CDR-L3 loop and likely support the loop conformation or stabilize the VH/VL interface. The significant effect of these mutations suggests that the paratope lies across both the VH and VL, encompassing at least CDR-H3 and CDR-L3.

While bispecific diabodies or bispecific single-chain Fvs are efficient at re-targeting cytotoxic T lymphocytes to tumor cells, they are cleared from circulation with a terminal half-life within a few hours due to their small size [16,34,35]. This rapid clearance can impact therapeutic administration, requiring constant infusion in the case of blinatumomab [35,36]. To extend circulating half-life, we engineered an Fc fusion DART molecule by replacing the E and K coils with human IgG1 Fc domains. Incorporation of an Fc increases molecular weight and extends the serum half-life through interaction with the neonatal Fc receptor FcRn [27]. In addition to half-life extension, Fc fusion frequently offers other benefits such as increasing recombinant protein expression, simplifying purification through Protein A affinity chromatography, and improving solubility and stability [37]. We have observed these improvements in the fusion of DART 35 and its variants to Fc, discussed below.

To replace the heterodimerization function of the E and K coils, the knobs-into-holes design strategy was employed, introducing a set of Fc mutations that favor heterodimerization over the normal Fc homodimerization [18,19]. Additional modifications were made to the Fc to prevent ADCC through FcγR binding and inadvertent activation of CD3 in the absence of target, using mutations in the lower hinge region that have previously been demonstrated to abrogate ADCC activity [28]. To identify the Fc fusion structure that supports optimal DART functional and biophysical properties, we generated eight Fc-DART variants of DART 35, with varying fusion linker lengths and hinge region lengths. While all LP DART proteins demonstrated an improvement in transient protein expression compared to the non LP-DART format (4–7-fold), an increase in HMMS was detected for all constructs following Protein A capture (33%–45% *vs.* 7% for the non LP-DART format). This increase in HMMS is commonly seen with Fc-fusion proteins and can be easily removed with conventional chromatography. Binding activity for all LP-DART proteins to both P-cadherin and CD3 showed a modest decrease compared to the non-Fc DART format (1.5–3-fold), as did T cell-directed cytotoxicity on tumor cells (2.2–8.6-fold). The linker-hinge design that demonstrated the lowest impact on binding and CTL activity, LP-DART 5, contained a single glycine separating the *C*-terminal serine from the VH domain and a truncated hinge region. We hypothesize that this short linker and truncated hinge minimize flexibility of the Fc domain and thereby reduce interference with antigen binding. When stably transfected into CHO cells, the affinity-optimized and Fc-engineered DART PF-06671008 (DART 153) was expressed at 1.3 grams/liter following Protein A purification. Using conventional bioprocessing chromatographic techniques, we were able to remove aggregates and inactive homodimer species, resulting in high purity (Table 5, Figure 6). The molecule exhibited antibody-like thermostability, with a T_m_1 value ≥ 68°C.

Pharmacokinetic properties of the anti-P-cadherin/anti-CD3 LP-DART protein were assessed in a human FcRn knock-in mouse model. Since the LP-DART molecule tested does not recognize mouse P-cadherin or mouse CD3, pharmacokinetics in this model were unaffected by target binding. The LP-DART protein exhibited a biphasic elimination curve with a clearance rate of 1.3 mL/h/kg and a terminal half-life of 105.7 h. Although not quite as long as some IgG, this increase to ~4.4 days from just a few hours for DART proteins without Fc [16] represents a major improvement in serum half-life and will allow for less frequent therapeutic administration compared to standard DARTs and BiTEs.

ELISA, Biacore, and KinExa analyses demonstrated that appending an Fc domain maintained adequate antigen binding properties. The equilibrium dissociation constant of PF-06671008 against cynomolgus monkey P-cadherin determined by Biacore was 0.521 nM *versus* 0.231 nM for the DART version of 153 (Table 5). Kinetic binding analysis with CHO cells over-expressing human P-cadherin using KinExA demonstrated an equilibrium dissociation constant of 0.176 nM *versus* 0.352 nM for cynomolgus monkey protein, indicating a similar level of binding between human and cynomolgus monkey P-cadherin.

Consistent with its high-affinity binding, PF-06671008 exhibited potent redirected T cell-mediated killing of P-cadherin-positive cell lines with a range of P-cadherin expression levels. The EC_50_ values of 3.1 pM for DU145 cells and 122.6 pM for HCT-116 cells tracked with their P-cadherin expression levels. Human PBMCs alone, which do not express P-cadherin, were not killed in the presence of PF-06671008, and a control DART capable of engaging CD3 on T cells (4420-LP) did not mediate redirected killing of target cells, suggesting there is a strict requirement for P-cadherin expression for activity. PF-06671008-mediated redirected target cell killing was associated with a concomitant dose- and target-dependent induction of cytokine release by human T cells, as represented by increased levels of TNF-α, IFN-γ, IL-10, IL-6, IL-4, and IL-2 in supernatants. Similar to cytotoxicity, the sensitivity to PF-06671008-mediated cytokine release (as reflected by lower EC_50_ values) and maximal levels of cytokine release (E_max_) induced by PF-06671008 showed lower EC_50_ and higher E_max_ values for cells with higher expression of P-cadherin.

PF-06671008 demonstrated potent *in vivo* tumor growth inhibition in a colorectal carcinoma tumor model. Treatment with PF-06671008 administered as a single agent IV once daily for four doses resulted in a dose-dependent inhibition of tumor growth when HCT-116 adenocarcinoma tumor cells were implanted SC in the presence of activated human T cells. Inhibition of tumor growth was observed at PF-06671008 doses ≥ 0.01 µg/kg, while no tumor growth was observed in mice treated with doses ≥ 1 µg/kg.

Clinical dosing of this class of T-cell re-directing therapy has been extraordinarily low due to a combination of potency and adverse events associated with cytokine release syndrome (CRS) [35], [36]. P-cadherin expression is upregulated in multiple tumor types, whereas healthy tissues that do express endogenous P-cadherin have been documented to express much lower levels [6]. Therefore targeting P-cadherin with this type of therapy may offer a reasonable therapeutic window at the expected low clinical dose range. Further investigation is underway to better understand the safety profile of PF-06671008.

## 4. Experimental Section

### 4.1. Enzyme-Linked Immunosorbent Assay (ELISA) to Measure Binding of scFv/DART Proteins to Recombinant Cadherin or CD3 Proteins

P-cadherin-Fc protein (R & D Systems, Minneapolis, MN) or P-cadherin His or negative control (E-cadherin-Fc and VE-cadherin-Fc; R & D Systems, Minneapolis MN) or CD3 epsilon-delta protein was coated overnight at 4 °C on 96-well Nunc Maxisorp plates (Thermo Fisher Scientific, Madison, CT, USA) at a concentration of 1 or 2 µg/mL in PBS. Plates were washed three times using PBS + Ca^2+^ and Mg^2+^ and blocked for 1 h at room temperature in 3% milk/PBS + Ca^2+^ and Mg^2+^. Samples prepared in block buffer were added to the blocked plates for 1 h at room temperature. Plates were washed three times with PBS + Ca^2+^ and Mg^2+^ prior to the addition of secondary antibody (either anti-human IgG-HRP 1:4000 (Southern Biotech, Birmingham, AL, USA) or anti His 1:2000 (Qiagen, Valencia, CA, USA). In some cases, prior to the addition of secondary antibody, plates were allowed to incubate in wash buffer overnight at 4 °C in an effort to detect improvements in off-rate. Plates were incubated for a further 1 h at room temperature and washed three times with PBS + Ca^2+^ and Mg^2+^. Signal was developed using TMB (SurModics, Eden Prairie, MN, USA), the reaction stopped with H_2_SO_4_, and the absorbance read at 450 nm.

### 4.2. Bi-Specific ELISA to P-Cadherin and CD3

Unpurified conditioned medium containing DART proteins as well as purified DART proteins were screened by ELISA for the ability to bind P-cadherin and CD3 simultaneously. Bispecific ELISA methods were previously described [17] with the following modifications: CD3 epsilon/delta heterodimer was coated as described above, followed by incubation of test sample diluted in block buffer (3% milk/PBS) for 1 h at room temperature. Plates were then treated with sequential addition of 2 µg/mL P-cadherin-Fc-His (R & D Systems, Minneapolis, MN) for 1 h in 3% milk/1% bovine serum albumin (BSA)/PBS + Ca^2+^ and Mg^2+^ and anti His-HRP at 1:2000 (Qiagen, Valencia, CA, USA) with intervening plate washing. Bound complex was detected with TMB, the reaction stopped with H_2_SO_4_, and the absorbance read at 450 nm.

### 4.3. Quantitative Flow Cytometry to Measure P-Cadherin Expression

The anti-P-cadherin monoclonal antibody PF-06671003 [20] was labeled with phycoerythrin with a fluorophore to protein ratio of 1:1 (eBioscience, San Diego, CA, USA) and used to measure P-cadherin expression by flow cytometry. Tumor cells (listed in Figure 2A) were added to assay plates at 10^6^ cells/mL and labeled with PF-06671008-PE in 100 μL/well of FACS buffer. The plates were incubated in the dark at 4 °C for 30 min and then 150 μL of FACS buffer was added to each well, followed by centrifugation at 311× *g* (1200 rpm) for 5 min. The supernatant was removed and the cell pellets were washed once with 200 μL/well FACS buffer. After centrifugation, the cell pellets were resuspended in 100 μL/well FACS buffer for cell event collection by FACS Calibur flow cytometer equipped with acquisition software CellQuest Pro Version 5.2.1 (BD Biosciences, San Jose, CA, USA). Data analysis was performed using Flowjo v9.3.3 software (Treestar, Inc., Ashland, OR, USA).

### 4.4. ELISA to Measure Binding of DART Proteins to P-Cadherin-Expressing Cells

NCI-H1650 cells expressing endogenous P-cadherin or parental CHO-DUKX cells were seeded at 4 × 10^4^ cells/well in a 96-well tissue culture plate on Day 1 and incubated at 37 °C/5% CO_2_ overnight until a confluent monolayer was observed. Cells were washed three times with PBS + Ca^2+^ and Mg^2+^and blocked for 1 h at room temperature with 3% milk/PBS + Ca^2+^ and Mg^2+^. Test samples diluted in block buffer were transferred to the plates and incubated for 1 h at room temperature. Plates were washed three times with PBS + Ca^2+^ and Mg^2+^ prior to the addition of secondary antibody (either anti-human IgG-HRP 1:4000 (Southern Biotech, Birmingham, AL, USA) or anti His 1:2000 (Qiagen, Valencia, CA, USA). Plates were incubated for a further 1 h at room temperature and washed four times with PBS + Ca^2+^ and Mg^2+^. Signal was developed using TMB, the reaction stopped with H_2_SO_4,_ and the absorbance read at 450 nm.

### 4.5. Control Anti-P-Cadherin/Anti-CD3 DART Generation

Positive (PF-DART) and negative (4420-DART) control were generated from PF-03732010, a monoclonal anti-P-cadherin antibody [20], and from an anti-fluorescein antibody, 4420 ([17,38], using methods previously described [16,17]. These DART constructs were engineered with the XR32 anti-CD3 epsilon antibody [39,40].

### 4.6. Selection of P-Cadherin-Specific scFv by Phage Display Using Naïve Library

ScFv that bind to the ECD of P-cadherin were identified following three rounds of selection using Pfizer’s in-house naïve phage display library. Selection strategies are outlined in Figure S1. The library is composed of VH and VL domains derived from non-immunized human donors, randomly paired and cloned into a phagemid vector upstream of C terminal His_6_ and c-Myc tags. Phage library rescue and phage selection were performed as previously described [41].

### 4.7. Cloning, Sequencing and Reformatting to DARTs

ScFv DNA was sequenced on both strands (Genewiz, Cambridge, MA, USA or Wyzer, Cambridge, MA, USA) as either unpurified bacterial glycerol stock or as purified plasmid using conventional methods. ScFv fragments demonstrating strong binding to P-cadherin or cell surface (>3× background or negative cell binding) were selected for reformatting as DARTs. DART design and cloning methods were previously described [16,17,40]. Methods for adapting novel P-cadherin scFv from Pfizer’s in-house naïve library and mutant libraries are as follows: Fragments were amplified by standard polymerase chain reaction (PCR) with primers annealing to human germline V and J sequences and incorporating restriction sites for DART cloning (BamHI/BspEI for VH and BssHII/BamHI for VL). Fragments were digested with corresponding restriction enzymes according to the manufacturer’s specifications (New England Biolabs, Ipswich, MA, USA). Anti-P-cadherin VH or VL fragments were gel-purified (Gel Purification Kit, Qiagen, Valencia, CA, USA) and ligated separately into Pfizer proprietary pre-digested mammalian expression vectors containing either the VL or VH, respectively, of anti-CD3 scFv as well as either of two alternative carboxy-terminal heterodimerization domains termed the E-coil and K-coil domains (Figure 1A).

### 4.8. Affinity Maturation Library Construction and Identification of Optimized scFv

Variants of anti-P-cadherin scFvs with increased affinity were isolated from mutagenized libraries using two approaches. The first approach focused on parental clone 35 using random mutagenesis of the heavy chain coupled with light chain shuffling. Random mutations were introduced into the VH domain clone 35 by error-prone PCR using the GeneMorph II kit according to the manufacturer’s protocol (Agilent Technologies, Santa Clara, CA, USA). Mutagenized VH pools were digested with BssHII and XhoI and sub-cloned into a pool of phagemid vectors containing approximately 1 x10^10^ variable light (VL) genes from non-immunized human donors.

Mutant phage libraries were rescued as described previously [41]. Two selection strategies were performed with these mutant libraries, including a two round solution-phase selection approach termed Hammer-hug [23] and a cell-based selection approach described as follows. Approximately 4 × 10^7^ cells not expressing P-cadherin (de-selection cells) were collected using cell dissociation buffer (PBS/5 mM EDTA) and washed twice with PBS. Cells were blocked with 3% milk/1% BSA/PBS for 1 h at 4 °C on a rotary mixer (20 rpm). Adherent cells expressing P-cadherin (capture cells) were plated 1 day prior at 1.2 × 10^6^ cells/6-well plate, grown overnight at 37 °C, then washed with PBS + Ca^2+^ and Mg^2+^ and subsequently blocked with 3% milk/1% BSA/PBS for 1 h at room temperature. De-selection cells were collected by centrifugation, re-suspended in blocked phage and incubated at 4 °C as before. De-selection cells were pelleted and the phage supernatant was transferred to the blocked capture cells and allowed to incubate for 2 h at room temperature. The capture cells were washed five times with cold PBS + Ca^2+^ and Mg^2+^/0.1% Tween 20 and ten times with cold PBS + Ca^2+^ and Mg^2+^. Phage were eluted by incubating the cells in 100 mM triethylamine (TEA) solution for 10 min at room temperature, then harvesting all cell and media from each well. Eluted phage were harvested in the supernatant following centrifugation of cells. Phage were then recovered by infecting an ER2738 *E. coli* host and rescued as previously described [41].

The second affinity optimization approach focused on parental clone 34, using a soft randomization approach targeting all CDRs except CDR-L2, as previously described [23]. Phage selections were split across two branches and over three rounds of increasing stringency, with branch 1 starting at 50 nM and branch 2 starting at 10 nM. Each branch decreased in target concentration 10-fold over each successive round. Phage selections and screening were performed as previously described [23,41].

### 4.9. Binding Affinity Analysis by Surface Plasmon Resonance

The Biacore 3000 and T-200 instruments (GE Healthcare, Piscataway, NJ, USA) were used to determine affinity and kinetics of recombinant human CD3 epsilon-delta, human P-cadherin-Fc, and mouse and cynomolgus monkey P-cadherin ECD proteins binding to DART protein. P-cadherin or human CD3 proteins were immobilized on CM5 Biacore sensor chips using routine amine coupling. The immobilization buffer was 10 mM acetate buffer pH 4.5 with 2.0 mM CaCl_2_. Surface densities ranged from ~75–300 RU. A non-derivatized flow cell was used as a reference surface. Two-fold titration series of DART samples in running buffer were prepared at concentrations ranging from 100 to 6.25 nM, and running buffer alone was included as a zero reference. Samples were injected in duplicate for 60 s at a flow rate of 30 µL/min across the flow cells using TBS-P+ (10 mM Tris pH 7.4, 150 mM NaCl, 2 mM CaCl_2_, 0.1 mg/mL BSA 0.05% P20) as running buffer. Dissociation of the DART proteins was monitored for 180 s followed by regeneration with a 10 second injection of 10 mM glycine-HCl pH 1.5 at 30 µL/min. The data were fit globally to a 1:1 Langmuir binding model using BIAevaluation software (GE Healthcare, Piscataway, NJ, USA).

### 4.10. Binding Affinity Analysis by KinExA

A Kinetics Exclusion Assay (KinExA) instrument (model 3200, Sapidyne, Boise, ID, USA) was used to compare the DART protein binding affinity to recombinant cynomolgus monkey P-cadherin and human P-cadherin expressed on the surface of CHO cells. Binding experiments were performed in PBS buffer pH 7.4 containing MgCl_2_, CaCl_2_ and 1 mg/mL BSA using PMMA beads (Sapidyne, Boise, ID, USA, catalog number 440176) at 4 °C for 2 h. Receptor binding concentration was measured using the SACy5 fluor at 13 time points in 500 µL volume.

### 4.11. Fc-DART Protein Engineering

For Fc fusion linker and hinge assessment, fragments were prepared as before and ligated separately into pre-cut mammalian expression vectors containing either the VL or VH of anti CD3 scFv, either of two modified human IgG1 Fc domains, and varying linker/hinge sequences listed in Table 4. These undigested expression vectors were synthesized at an external vendor and digested with appropriate restriction enzymes as described above prior to ligation. The “knob” Fc variant was constructed by replacement of a small amino acid with a larger one, e.g., T366W. The “hole” Fc variant was constructed by replacement of a large residues with a smaller ones e.g., T366S, L368A, and Y407V. Additionally, two cysteines (Y349C on the knob chain and S354C on the hole chain) were added for the introduction of a disulfide bond for added stability. All constructs were confirmed by DNA sequencing and transiently transfected into FreeStyle™ 293 HEK cells (Life Technologies, Grand Island, NY, USA) according to the manufacturer’s method and expressed over 5–7 days.

### 4.12. Stable CHO Cell Line Development and Expression

The two chains of PF-06671008 were cloned into a mammalian expression vector containing dual promoters and engineered for integration into a single recombination site engineered into the host CHO cell genome. The PF-06671008-Knob construct was cloned into the first cloning site, referred to as cassette 1. The PF-06671008-Hole construct was cloned into the second cloning site, cassette 2. The resulting plasmid was stably transfected into CHO host cells. A 10 L working volume Wavebag (GE Healthcare, Piscataway, NJ, USA) was seeded with 1.5 L cell culture in 8 L of Pfizer’s proprietary cell culture medium. The culture was grown at a temperature of 36.5 °C in a 5% CO_2_ environment. Cultures were fed with a proprietary feed at various time points. Conditioned medium was harvested on day 12 by filtration through a 20” 5 µm Pall Profile II filter (Port Washington, NY, USA) and a 10” 0.22 µm Pall Supor filter (Port Washington, NY, USA).

### 4.13. Purification of PF-06671008

Clarified conditioned medium was purified using conventional chromatographic techniques. The final pool was analyzed by OD280 (NanoDrop™, Thermo Fisher Scientific, Madison, CT), SDS-PAGE (BioRad Laboratories, Hercules, CA, Stain Free 4%–15%), analytical SEC (TSK G3000), and analytic HIC. CHO host-cell protein and Protein A leaching were quantified using ELISA kits following the manufacturer’s protocol (Cygnus Technologies, Southport, NC, USA). Analytical SEC was performed on an Agilent 1200 series HPLC (Agilent Technologies, Santa Clara, CA, USA) using either a Superdex200 10/30 column (GE Healthcare, Piscataway NJ, USA) or a TSKgel G3000SWxl column (Tosoh Bioscience, King of Prussia, PA, USA) according to the manufacturer’s protocol. An analytical HIC-HPLC assay was used to assess protein heterogeneity. Using an Agilent Infinity 1290 UHLPC (Agilent Technologies, Santa Clara, CA, USA), approximately 20 to 30 µg of protein was injected at a flow rate of 1 mL/min and protein was detected by absorption at 280 nm.

### 4.14. Mass Spectrometric Analysis

Purified LP-DART protein was analyzed by liquid chromatography/mass spectrometry (LC/MS) analysis on an Agilent 1100 capillary HPLC coupled with Water Xevo G2 Q-TOF mass spectrometer (Santa Clara, CA, USA). The analytes were loaded onto a ZORBAX Poroshell 300SB C8 column (Agilent Technologies, Santa Clara, CA, USA, 0.5 mm × 75 mm, maintained at 80 °C) with 0.1% formic acid, and eluted using a gradient of 20%–40% buffer B (80% acetonitrile, 18% 1-propanol, 2% water with 0.1% formic acid) at a flow rate of 20 µL/min over 5.5 min. Mass spectrometric detection was carried out in positive, sensitivity mode with capillary voltage set at 3.3 kV. Data analyses were performed with MaxEnt 1 function in MassLynx (Waters, Milford, MA, USA).

### 4.15. Differential Scanning Calorimetry

Purified DART samples were diluted in PBS to 1 mg/mL in a volume of 400 µL. PBS was used as a buffer blank in the reference cell. Samples were dispensed into the sample tray of a MicroCal VP-Capillary differential scanning calorimeter (DSC) with Autosampler (GE Healthcare, Piscataway, NJ, USA). Samples at either 0.3 mg/mL or 1 mg/mL were equilibrated for 5 min at 10 °C and then scanned up to 110 °C at a rate of either 60 °C/h or 100 °C/hr. A filtering period of 16 s was selected. Raw data were baseline-corrected, and the protein concentration was normalized. Origin Software 7.0 (OriginLab Corporation, Northampton, MA, USA) was used to fit the data to an MN2-State Model with an appropriate number of transitions.

### 4.16. Epitope Mapping of DART Proteins on the Human P-Cadherin Extracellular Domain

To identify the binding epitope of the anti-P-cadherin/anti-CD3 DART molecules, soluble P-cadherin ECD-Fc fusion protein constructs were generated. Each P-cadherin-Fc construct comprised a signal peptide, pro-peptide and a P-cadherin ECD subdomain region (either ECD1, ECD1–2, ECD1–3, ECD1–4, or ECD1–5) genetically fused to the hinge and CH2 and CH3 domains of human IgG1 via cleavable linker, as shown in Figure 4. All constructs were confirmed by DNA sequencing and transiently transfected into FreeStyle™ 293 HEK cells (Life Technologies, Grand Island, NY, USA) according to the manufacturer’s method and expressed over 5–7 days. For enhanced processing of the pro-peptide, an expression vector containing the PACE cleavage enzyme was co-transfected along with the P-cadherin-containing vector. Purified protein was characterized for purity and activity by binding ELISA using commercially-available anti-human P-cadherin monoclonal and polyclonal antibodies.

### 4.17. Crystallography of Coil-Less DART 35

For crystallization trials, a modified version of DART 35 was generated with either a His_6_ tag or a FLAG tag at the *C*-terminus of each DART subunit chain to aid in purification (Figure 5C). The constructs were transiently transfected into FreeStyle™ 293 HEK cells as described above. 10 mL anti-FLAG M2 resin (Sigma, St. Louis, MO, USA) was pre-equilibrated in TBS and allowed to batch-bind in 1.4 L conditioned medium for 2 h at 4 °C. The resin was then collected and packed into a column for anti-FLAG M2 chromatography, washed to baseline with TBS (20 CVs) and initially eluted with 0.1 M FLAG peptide buffer and finally eluted with 0.1 M glycine pH 3.0. The eluted protein was immediately neutralized with 10% 1.0 M Tris pH 8.0. Fractions with the highest purity were pooled, and the eluted protein was then further purified by Ni-NTA chromatography. Five mL of Ni-NTA resin pre-equilibrated in TBS was allowed to batch-bind to the protein for 1 h at 4 °C on an orbital mixer. The resin was then collected and placed in an Applied Biosystems column (Life Technologies, Grand Island, NY, USA). Resin was first washed to baseline with Buffer A (50 mM sodium phosphate, 300 mM sodium chloride pH 8.0), then with five CVs of Buffer A supplemented with 20 mM imidazole and finally eluted with Buffer A + 250 mM imidazole. Fractions with the highest purity were then pooled and further purified by size exclusion chromatography using a Superdex200 column (GE Healthcare, Piscataway, NJ, USA) and stored in TBS.

The purified HIS/FLAG-DART was concentrated to 9.6 mg/mL in a protein solution containing TBS. The crystals were obtained by hanging-drop vapor-diffusion method from a condition containing 15% PEG 8K and 0.5 M lithium sulfate. The hexagonal plate-like crystals had symmetry consistent with trigonal space group P321 with cell parameters a = b = 142.81 Å; c = 62.69 Å and with one coil-less DART molecule in the crystallographic asymmetric unit. The crystals were cryo-protected using a reservoir solution containing 25% ethylene glycol and were flash frozen in liquid nitrogen. A data set to a 2.0 Å resolution was collected from a single frozen crystal at IMCA beamline 17-ID at the Argonne National Laboratory (Lemont, IL, USA, APS). The data were processed and scaled using autoPROC (Global Phasing Ltd., Cambridge, UK) and SCALA (École Polytechnique Fédérale de Lausanne, Lausanne, Switzerland). The final data set was 96.8% complete with average redundancy of 9.9 and with Rsym of 14.2%.

The structure was solved by molecular replacement with PHASER starting with the single chain Fv fragment models prepared from the Brookhaven PDB entry code, 1MOE. The solution was obtained by searching for each of the four subunits of the DART molecule separately. Several iterative rounds of manual adjustment and model rebuilding using COOT and crystallographic refinement using autoBUSTER yielded the final DART model with a crystallographic Rwork of 17.6% and Rfree of 20.5%, where Rwork= ||Fobs| − |Fcalc||/|Fobs| and Rfree is equivalent to Rwork, but calculated for a randomly chosen 5% of reflections omitted from the refinement process.

### 4.18. Isolation of PBMCs and T Cells from Human Whole Blood

Peripheral blood mononuclear cells (PBMCs) from healthy human donors were isolated from whole blood using Ficoll gradient centrifugation. Whole blood was diluted 1:1 with sterile Dulbecco’s phosphate buffered saline (DPBS). The diluted blood (35 mL) was layered onto 15 mL of Ficoll-Paque Plus in a 50 mL tube and the tubes were centrifuged at 400× *g* (1320 rpm) for 30 min with the brake off. The buffy coat layer between the two phases was collected into 50 mL tubes and centrifuged at 600× *g* (1620 rpm) for 5 min. The supernatant was discarded and the cell pellet was washed three times with DPBS by centrifuging the tubes at 600× *g* (1620 rpm) for 5 min. Viable cell count was determined using Trypan Blue dye to exclude non-viable cells. The PBMCs were resuspended in complete culture medium (RPMI 1640, 10% FBS, 1% pen/strep) and incubated at 37 °C with 5% CO_2_ overnight or were further processed to isolate human CD4+ or CD8+ T cells. T cells were isolated from PBMCs using the untouched human CD4+ T cell isolation kit or untouched human CD8+ T cell isolation kit (Life Technologies, Grand Island, NY, USA) according to the manufacturer’s instructions. After isolation, the T cells were resuspended in complete culture medium and incubated at 37 °C with 5% CO_2_ overnight.

### 4.19. CTL Cytotoxicity Assay (LDH Release Assay)

The CytoTox 96^®^ Non-Radioactive Cytotoxicity Assay Kit (Promega, Madison, WI, USA) was used to measure cytotoxicity by quantitating the enzymatic activity of lactate dehydrogenase (LDH) released from lysed cells. The three target cancer cell lines were harvested by detaching cells with 0.25% Trypsin-EDTA solution and collected by centrifugation at 311× *g* (1200 rpm) for 5 min. The cells were washed once by DPBS and resuspended in assay medium (RPMI 1640 no phenol red, 10% FBS, 1% pen/strep). After counting and confirmation of viability (>85%), the cells were diluted in assay medium to a density of 4 × 10^5^ cells/mL. 50 μL of the diluted cell suspension was added to a 96-well, U-bottom cell culture plate (BD Falcon). For each treatment, duplicate wells were included.

The effector cells (human PBMCs or purified human CD4+/CD8+ T cells) were washed once with assay media and resuspended in assay media at the appropriate cell density depending on the effector and target (E:T) cell ratio used in the assay. Effector cells (100 µL) were added to each well of the plate containing 50 µL target cancer cells. Test DART proteins were initially diluted to 4-times the highest concentration to be added to the assay plate and serially diluted 4-fold. 50 µL/well of the dilutions were added to the plate containing 100 μL effector cells/well and 50 μL target cells/well.

Three sets of controls to measure target cell spontaneous release (SR), antibody independent cellular cytotoxicity (AICC), and target cell maximal release (MR) were set up as follows: (1) SR: 50 µL target cells and 150 µL assay media without test molecules; (2) AICC: 50 µL target cells, 100 µL effector cells, and 50 μL assay media without test molecules; and (3) MR: 50 μL target cells and 120 μL assay media without test molecules to which lysis solution was added at the end of the experiment to determine maximal LDH release.

Plates were incubated at 37 °C with 5% CO_2_ for 24 to 48 h as indicated. Following incubation, 30 μL/well of 10× lysis solution was added to the maximum release wells, mixed by pipetting three times, and plates were incubated for 10 min to completely lyse the target cells. The plates were centrifuged at 311× *g* (1200 rpm) for 5 min and 40 µL/well of supernatant were transferred to a flat-bottom ELISA plate and 40 µL of LDH substrate solution was added to each well. Plates were incubated for 10–20 min at room temperature in the dark and then 40 µL of stop solution was added. The optical density (OD) was measured at 490 nm within 1 h on a Victor2 Multilabel plate reader. The percentage of cytotoxicity was calculated as described below and dose-response curves were generated using GraphPad Prism5 or 6 software by curve fitting the cytotoxicity values to the sigmoidal dose-response function.

Specific cell lysis was calculated from OD data using the following formula:

Cytotoxicity (%) = 100 × (OD of Sample − OD of AICC)/(OD of MR − OD of SR)
(1)

### 4.20. Determination of Cytokines in Supernatant

An ELISA was used to measure the levels of six cytokines in the supernatants (IFN-γ, TNF-α, IL-10, IL-6, IL-4, and IL-2). Cytokine detection kits were purchased from R & D Systems. The ELISA plate (Thermo Scientific, Madison, CT, USA) was coated with capture reagent from the kit diluted in 1× DPBS at the coating concentration suggested by the manufacturer and incubated at room temperature overnight. The next day the coated plate was washed with wash buffer containing 1× DPBS and 0.1% Tween20. The plate was then blocked with 200 µL/well of sample diluent containing 1× DPBS and 1% BSA for 1 h at room temperature and then washed. During the 1-h blocking step, test samples and standard samples (for generating standard curves) were prepared. After the blocking step, 100 µL/well of standard samples and test samples were added to the plate, followed by incubation for 2 h. The plate was incubated for an additional 2 h after 100 µL/well of diluted detecting reagent was added. Then the plate was washed again and 100 µL/well of streptavidin-horseradish peroxidase (SA-HRP) solution was added followed by incubation for 20 min. After washing, 100 µL/well of TMB One Component HRP Microwell Substrate was added followed by incubation for 15 to 20 min in the dark. The reaction was stopped by adding 1% sulfuric acid. The OD was measured at 450 nm within 15 min on a Victor 2 Multilabel plate reader. The data were exported as Microsoft Excel format for data analysis. The standard curves were generated using SoftMax Pro Version 5.4 (Molecular Devices) by fitting the data from each cytokine standard to a 4-parameter variable weight function with concentration of cytokine (x) as the independent variable and OD response (y) as the dependent variable:

y = (A − D)/(1 + (x/C)^B) + D
(2)

In this expression, A refers to the left (lower) asymptote, D refers to the right (upper) asymptote, C refers to the cytokine concentration that produces 50% of the maximum response, and B is a scale parameter related to the shape and steepness of the curve. The curve fit is performed using the variable weight for each point option in SoftMax Pro with the formula 1/Y^2^.

The cytokine concentration was calculated from the 4-parameter curve using the following SoftMax command:

InterpX(Plot#N@Graph#N@Experiment#N,Values)
(3)

where X is the concentration of the analyte, Plot #N refers to the standard curve on Graph #N for Plate #N. Values are the mean OD value for the respective concentration of the cytokine.

### 4.21. Pharmacokinetic Property Assessment in a Human FcRn Knock-in Mouse Model

Female B6.Cg-Fcgrttm1Dcr Tg(CAG-FCGRT)276Dcr/DcrJ mice (Jackson Laboratories Inc., ID 004919, Bar Harbor, ME, USA) were dosed intravenously with anti-P-cadherin/anti-CD3 LP-DART at 2 mg/kg. Mice (*n* = 3/time point) were bled prior to dosing and at 5 min, 4 h, and on day 1, 2, 3, 7, 14 and 22. Serum was prepared and frozen at −80 °C until assayed.

Assay plates (MaxiSorp 96-well, Nunc, Rochester, NY, USA) were coated with a custom polyclonal goat anti-CD3 antibody overnight at 4 °C. Plates were washed three times using PBS + Ca^2+^ and Mg^2+^ and blocked for 1 h at room temperature in 0.5% BSA. Plates were then incubated with diluted test samples, a P-cadherin LP-DART calibration standard and appropriate controls. Plates were washed as before pre-, during and post sequential 1 h incubations with goat anti-human IgG Fc-biotin (Thermo Scientific, Madison, CT, USA) and streptavidin-horseradish peroxidase (SA-HRP; Thermo Scientific, Madison, CT, USA). Signal was developed using TMB (SurModics, Eden Prairie, MN, USA) and the reaction stopped with H2SO4. Plates were read at OD450 nm using a microplate reader (SpectraMax M2e, Molecular Device, Sunnyvale, CA, USA). The calibration standard curve was generated with the standard calibrators OD signals in the four-parameter logistic model using SoftMax Pro software (Version 5.4, Molecular Devices). Concentrations were determined from the interpolation of the samples’ OD signal data with the equation describing the standard curve. The lower limit of quantitation (LLOQ) for this assay was estimated to be 9.75 ng/mL. PK parameters were calculated using the WinNonlin non-compartment analysis (NCA) model.

### 4.22. In Vivo Tumor Growth Inhibition by PF-06671008

Human T cells were isolated from heparinized whole blood according to the manufacturer’s protocol provided in the RosetteSep T cell isolation kit. The purified T cells were subsequently activated by exposing the cells to anti-CD3 (OKT-3; 1 µg/mL) and anti-CD28 (66 µg/mL) antibodies for a period of 48 h. Following stimulation, the cells were grown in RPMI 1640 medium with 10% FBS and 1% penicillin/streptomycin in the presence of IL-2 (7.6 ng/mL) for up to 3 weeks. The HCT-116 cells were maintained *in vitro* in RPMI 1640 medium with 10% FBS and 1% penicllin/streptomycin. The human T cells and tumor cells were combined at a ratio of 1:5 (1 × 10^6^ and 5 × 10^6^ cells, respectively) and suspended in 200 µL of sterile Ham’s F12 and injected SC on Day 0.

Vehicle control (sterile saline containing 0.5% BSA), PF-06671008, or 4420-hXR32-LP control DART was administered IV via tail vein injections (100 µL) once daily for 4 days on Days 0, 1, 2, and 3 (see Table 7). Individual animal weights were recorded twice weekly beginning at the time of tumor cell injection until study completion. Animals were observed twice weekly for general moribundity and daily for mortality. Animal deaths were to be assessed as drug-related or technical based on factors including gross observation and weight loss; animal deaths were to be recorded daily. Individual tumor dimensions (length × width) were measured using calipers and were recorded periodically throughout the study beginning on Day 7 and continuing through study completion. Tumor volume was estimated as follows:

Tumor Volume (mm^3^) = length × width^2^/2 (4)

Animals that died for any reason were to be censored from the data calculations at the time of death. Tumor growth inhibition (TGI) values were calculated for each group containing treated animals using the formula:

(5) $$1-\frac{\text{Mean Final Tumor Volume }\left(\text{Treated}\right)-\text{ Mean Initial Tumor Volume }\left(\text{Treated}\right)}{\text{Mean Final Tumor Volume }\left(\text{Control}\right)-\text{ Mean Initial Tumor Volume }\left(\text{Control}\right)}\times 100$$

Individual mice lacking palpable tumors were classified as undergoing a complete response (CR). Statistical analyses were carried out between treated and control groups comparing tumor volumes. For these analyses, a two-way analyses of variance (ANOVA) followed by a Bonferroni post-test were employed. All analyses were performed using GraphPad Prism software (Version 6.03). Weight and tumor data from individual animals that died for any reason were to be censored from analysis (at the time of death). However, tumor data from animals reporting partial or complete responses were included in these calculations.

## 5. Conclusions

As more bispecific antibodies are developed and clinically investigated, the need for an improved format addressing the known shortcomings of these proteins becomes even more important [10]. Here, we have generated a stable, high-affinity anti-P-cadherin/anti-CD3 bispecific DART molecule with extended half-life that exhibits potent *in vivo* efficacy. Furthermore, we have demonstrated antibody-like manufacturability properties with high expression levels and utilized a protein purification process incorporating conventional, industry-standard techniques resulting in a highly pure, stable final product. PF-06671008 is a promising new therapeutic candidate for the treatment of solid tumors expressing P-cadherin. Additional studies are ongoing to better understand the safety and efficacy of this extended half-life DART protein.

## Figures and Tables

**Table 1:** Summary table of DART equilibrium dissociation constants (K_D_) to cynomolgus monkey P-cadherin and human CD3, ELISA cell binding EC_50_ values to NCI-H1650 and control P-cadherin-negative cells, and CTL-directed of NCI-H1650 adenocarcinoma cells by anti-P-cadherin/anti-CD3 DART proteins. * = samples were tested on a separate date with different donor effector cells. NB = no binding detected; ND = EC_50_ not determined; NR = no response.

DART ID	Equilibrium Dissociation Constant (K_D_) to Cyno P-cadherin (±SD) (nM)	Equilibrium Dissociation Constant (K_D_) to Human CD3 (±SD) (nM)	NCI-H1650 Cell Binding ELISA EC_50_ (nM)	CHO Parental Cell Binding ELISA EC_50_ (nM)	NCI-H1650 CTL Assay 48 h 10:1 EC_50_ (nM)
33 DART	46.4 ± 4.1	18.25 ± 0.55	60.1	NB	1.246
34 DART	129.25 ± 31.75	21.85 ± 0.95	67.95	NB	1.888
35 DART	43.4 ± 0.1	23.15 ± 0.45	24.87	NB	0.1042
PF DART	2.12 ± 0.71	6.80 ± 2.81	19.69	NB	0.0463
20 DART	2.03 ± 0.07	5.19 ± 0.35	~114.7	NB	ND
30 DART	1.37 ± 0.11	13.6 ± 3.49	141.9	NB	NR *

**Table 2:** Alignment of Amino Acid Sequences of anti-P-cadherin number 35 with variable light (VL)1 Germline 1b.366F5/DPL5...+. and variable heavy (VH)1 Germline DP-14/V1-18+. Star symbol represents identical sequence homology; Dash represents CDR3 region not defined in germline sequence according to Immunogenetics (IMGT) database.

Sequence Name	Amino Acid Sequence
**No. 35**	**QSVLTQPPSVSAAPGQKVTISCSGSSSNIGNNYVSWYQQLPGTAPKLLIYDNNKRPSGIP**
**DPL5**	**QSVLTQPPSVSAAPGQKVTISCSGSSSNIGNNYVSWYQQLPGTAPKLLIYDNNKRPSGIP**
	* * * * * * * * * * * * * * * * * * * * * * * * * * * * * * * * * * * * * * * * * * * * * * * * * * * * * * * * * * * *
**No. 35**	**DRFSGSKSGTSATLGITGLQTGDEADYYCGTWDSSLSAVVFGGGTKLTVL**
**DPL5**	**DRFSGSKSGTSATLGITGLQTGDEADYYCGTWDSSLSA- - - - - - - - - - - - **
	* * * * * * * * * * * * * * * * * * * * * * * * * * * * * * * * * * * * * *
**No. 35**	**EVQLVQSGAEVKKPGASVKVSCKASGYTFTSYGISWVRQAPGQGLEWMGWISAYNGNTNY**
**DP14**	**QVQLVQSGAEVKKPGASVKVSCKASGYTFTSYGISWVRQAPGQGLEWMGWISAYNGNTNY**
	- * * * * * * * * * * * * * * * * * * * * * * * * * * * * * * * * * * * * * * * * * * * * * * * * * * * * * * * * * * *
**No. 35**	**AQKLQGRVTMTTDTSTSTAYMELRSLRSDDTAVYYCATIDTASAFDIWGQGTMVTVSS**
**DP14**	**AQKLQGRVTMTTDTSTSTAYMELRSLRSDDTAVYYCAR- - - - - - - - - **
	* * * * * * * * * * * * * * * * * * * * * * * * * * * * * * * * * * * * * -

**Table 3:** Alignment of complementarity determining region (CDR) sequences with equilibrium dissociation constants and cytotoxicity EC_50_ values of affinity optimized anti-P-cadherin DARTs compared to parental DARTs.

DART	VL CDR1	VL CDR2	VL CDR3	VH CDR1	VH CDR2	VH CDR3	Cyno P-cad Biacore K_D_ (±SD) (nM)	CD3 Biacore K_D_ (±SD) (nM)	CTL HCT116 48-h 10:1 EC_50_ (nM)
34	SGSSSNIGNNYVS	DNNKRPS	GTWDSSLSAWV	SYGIS	WISAYNGNTNYAQKLQG	IDTASAFDI	129.25 ± 31.75	21.85 ± 0.95	3.83
35	SGSSSNIGNNYVS	DNNKRPS	GTWDSSLSAVV	SYGIS	WISAYNGNTNYAQKLQG	IDTASAFDI	43.40 ± 0.100	23.15 ± 0.45	0.2051
153	SGSSSNIGNNYVS	DNNKRPS	GTWDSSLSGVV	SYGIS	WISAYNGNTNYAQKLQG	IDTANAFGI	0.231 ± 0.017	15.65 ± 1.75	0.0005
154	SGSSSNIGNNYVS	DNNKRPS	GTWDSSLSSYV	SYGIS	WISAYNGNTNYAQKLQG	IDTATAFDI	3.930 ± 0.91	17.10 ± 0.90	0.0166
165	SGSSSNIGNNYVS	DNNKRPS	GTWDSSLSAYV	SYGIS	WISAYNGNTNYAQKLQG	IDTANAFDI	0.370 ± 0.087	14.90 ± 2.80	0.0015
177	SGSSSNIGNNYVS	DNNKRPS	GTWDSSLSAYV	SYGIS	WISAYNGNTNYAQKLQG	IDTANAFDI	0.483 ± 0.082	19.95 ± 6.55	0.0007
178	SGSSSNIGNNYVS	DNNKRPS	GTWDSSLSAVV	SYGIS	WISAYNGNTNYAQKLQG	IDTANAFDI	1.310 ± 0.14	23.10 ± 1.10	0.0018
179	SGSRSNIGNNYVS	DSNKRPS	GTWDSSLSSWV	SYGIS	WISAYNGNTNYAQKLQG	IDTANAFDI	4.165 ± 0.26	25.75 ± 9.25	0.0432
180	SGSSSNIGNNYVS	DNNKRPS	GTWDSSLSAYV	SYGIS	WISAYNGNTNYAQKLQG	IDTANAFDI	0.965 ± 0.019	15.9 ± 1.00	0.0018
281	SGSSSNIGNNYVS	DNNKRPS	GTWDSSLSAWV	SYGIS	WISAYNGNTNYAQKLQG	INAPNNFDI	NT	NT	NT

Red coloring indicates mutations away that differ from the parental DART #35; Blue coloring indicates a conserved residue in CDRL3 in parental DART #34.

**Table 4:** Biophysical and functional properties of LP-DART proteins. The optimal format for the Fc-containing DART was determined by varying the linker length (between diabody and Fc) and swapping the orientations of variable domains with corresponding knob and hole Fc domains. Linker sequences preceding the hinge sequence CPPCP are shown in bold. LP-DARTs were transiently expressed and evaluated for expression level, purity following affinity capture, equilibrium binding constants to cynomolgus P-cadherin and human CD3 epsilon, and CTL cytotoxicity. Capture Yield = total DART protein yield following Protein A capture step, measured in mg/L; % Agg Post capture = percentage of aggregation or HMMS following Protein A capture; Cyno-Pcad K_D_ (nM) = equilibrium dissociation constant measured by Biacore against cynomolgus monkey P-cadherin captured on the chip; CD3 K_D_ (nM) = equilibrium dissociation constant measured by Biacore against human CD3 captured on the chip; T cell Cytoxicity (nM) = redirected cytotoxic T lymphocyte killing EC_50_ value on NCI-H1650 cells incubated with resting T cells for 18 h.

Property	LP-DART #1	LP-DART #2	LP-DART #3	LP-DART #4	LP-DART #5	LP-DART #6	LP-DART #7	LP-DART #8	35 DART
First Chain	CD3VLx P-CAD 35VH-knob	CD3VLx P-CAD 35VH-knob	CD3VLx P-CAD 35VH-knob	CD3VLx P-CAD 35VH-knob	CD3VLx P-CAD 35VH-hole	CD3VLx P-CAD 35VH-hole	CD3VLx P-CAD 35VH-hole	CD3VLx P-CAD 35VH-hole	-
Second Chain	P-CAD 35VLx CD3VH-hole	P-CAD 35VLx CD3VH-hole	P-CAD 35VLx CD3VH-hole	P-CAD 35VLx CD3VH-hole	P-CAD 35VLx CD3VH-knob	P-CAD 35VLx CD3VH-knob	P-CAD 35VLx CD3VH-knob	P-CAD 35VLx CD3VH-knob	-
Linker	G CPPCP	GGTGG CPPCP	G EPKSS DKTHTCPPCP	GGTGGG EPKSS DKTHTCPPCP	G CPPCP	GGTGG CPPCP	G EPKSS DKTHTCPPCP	GGTGGG EPKSS DKTHTCPPCP	-
Capture Yield (mg/L)	24	23	35	28	20	24	27	34	5
% Agg Post capture	40%	37%	33%	39%	33%	40%	40%	45%	7%
Cyno-Pcad K_D_ (nM)	108.0	73.5	85.7	128.0	62.1	86.6	96.6	104.0	42.8
CD3 K_D_ (nM)	32.9	47.9	47.2	55.1	30.2	42.1	49.1	55.6	17.1
T cell Cytoxicity EC_50_ (nM)	0.79	0.92	1.60	2.45	0.74	1.29	2.85	2.27	0.33

**Table 5:** Functional and biophysical properties of PF-06671008. * Due to complexity in the dissociation rates, the K_D_ values could not be accurately determined. This may be caused by heterogeneity of the protein. Binding analyses performed by KinExA and Biacore demonstrated that PF-06671008 bound to human (cell surface) and cynomolgus monkey P-cadherin (recombinant protein) with similar affinities.

Expression Yield Post Protein A Capture	1.3 g/L
T_m_1 determined by DSC (°C)	≥68
EC_50_ binding to human P-cadherin ECD protein (nM) (ELISA)	0.92
EC_50_ binding to human CD3 protein (nM) (ELISA)	4.37
EC_50_ binding to NCI-H1650 cells (nM) (ELISA)	0.593
EC_50_ binding to CHO-parental cells (nM) (ELISA)	NB
Association rate constant to cyno P-cadherin (k_a_) (M^−1^·s^−1^) (Biacore)	4.37 × 10^5^
Dissociation rate constant to cyno P-cadherin k_d_ (s^−1^) (Biacore)	2.05 × 10^−4^
Biacore equilibrium dissociation constant to cyno P-cadherin (K_D_) (±SD) (nM)	0.521 ± 0.162
Biacore equilibrium dissociation constant to hu P-cadherin (K_D_) (nM)	>1 *
Biacore equilibrium dissociation constant to mu P-cadherin (K_D_) (nM)	NB
Biacore equilibrium dissociation constant to hu CD3 (K_D_) (±SD) (nM)	11.5 ± 0.9 **
KinExa equilibrium dissociation constant to cyno P-cadherin (K_D_) (nM)	0.352
KinExa equilibrium dissociation constant to CHO-hu P-cadherin+ cells (K_D_) (nM)	0.176

** Anti-CD3 domain was changed to a higher affinity version, differing by three amino acids. NB = no binding detected.

**Table:** A

CTL killing EC_50_	DU145 CTL (EC_50_, pM)	HCT-116 CTL (EC_50_, pM)	PBMC Alone CTL (EC_50_, pM)
PF-06671008	3.1	122.6	NR

**Table:** B

**Target Cell Line**	**Cytokine EC_50_ (ng/mL)**					
	**IFN-γ**	**TNF-α**	**IL-10**	**IL-6**	**IL-4**	**IL-2**
DU145	0.36	0.6	0.37	0.22	3.72	2.67
HCT116-Luc	28.68	18.24	21.24	6.98	5.43	6.89
PBMC Alone	NR	NR	NR	NR	NR	NR
**Target Cell Line**	**Cytokine E_max_ (pg/mL)**					
	**IFN-γ**	**TNF-α**	**IL-10**	**IL-6**	**IL-4**	**IL-2**
DU145	13513	7877	11871	1915	376	10225
HCT116-Luc	9881	5222	9231	697	154	694
PBMC Alone	NR	NR	NR	NR	NR	NR

**Table 7:** Study Design for HCT116 Tumor Model.

Treatment	Dose (µg/kg)	Route/Schedule	Number of Animals	Cell Implant(s)
Vehicle	0	IV/QDx4	8	HCT116 (5 × 10^6^)
PF-06671008	100	IV/QDx4	8	T Cells (1 × 10^6^) + HCT116 (5 × 10^6^)
PF-06671008	10	IV/QDx4	8	T Cells (1 × 10^6^) + HCT116 (5 × 10^6^)
PF-06671008	1	IV/QDx4	8	T Cells (1 × 10^6^) + HCT116 (5 × 10^6^)
PF-06671008	0.1	IV/QDx4	8	T Cells (1 × 10^6^) + HCT116 (5 × 10^6^)
PF-06671008	0.01	IV/QDx4	8	T Cells (1 × 10^6^) + HCT116 (5 × 10^6^)
4420-hXR32-LP	100	IV/QDx4	8	T Cells (1 × 10^6^) + HCT116 (5 × 10^6^)
Vehicle	0	IV/QDx4	8	T Cells (1 × 10^6^) + HCT116 (5 × 10^6^)

## Supplementary Materials

The following are available online at http://www.mdpi.com/2073-4468/5/1/6#supplementary.

## References

[1] Cheung L.W., Leung P.C., Wong A.S. (2010). Cadherin switching and activation of p120 catenin signaling are mediators of gonadotropin-releasing hormone to promote tumor cell migration and invasion in ovarian cancer. Oncogene.

[2] Paredes J., Stove C., Stove V., Milanezi F., van Marck E., Derycke L., Mareel M., Bracke M., Schmitt F.C. (2004). P-cadherin is up-regulated by the antiestrogen ICI 182,780 and promotes invasion of human breast cancer cells. Cancer Res..

[3] Ribeiro A.S., Albergaria A., Sousa B., Correia A.L., Bracke M., Seruca R., Schmitt F.C., Paredes J. (2010). Extracellular cleavage and shedding of P-cadherin: A mechanism underlying the invasive behaviour of breast cancer cells. Oncogene.

[4] Taniuchi K., Nakagawa H., Hosokawa M., Nakamura T., Eguchi H., Ohigashi H., Ishikawa O., Katagiri T., Nakamura Y. (2005). Overexpressed P-cadherin/CDH3 promotes motility of pancreatic cancer cells by interacting with p120ctn and activating rho-family GTPases. Cancer Res..

[5] Hardy R.G., Tselepis C., Hoyland J., Wallis Y., Pretlow T.P., Talbot I., Sanders D.S.A., Matthews G., Morton D., Jankowski J.A.Z. (2002). Aberrant P-cadherin expression is an early event in hyperplastic and dysplastic transformation in the colon. Gut.

[6] Imai K., Hirata S., Irie A., Senju S., Ikuta Y., Yokomine K., Harao M., Inoue M., Tsunoda T., Nakatsuru S. (2008). Identification of a novel tumor-associated antigen, cadherin 3/P-cadherin, as a possible target for immunotherapy of pancreatic, gastric, and colorectal cancers. Clin. Cancer Res..

[7] Paredes J., Albergaria A., Oliveira J.T., Jeronimo C., Milanezi F., Schmitt F.C. (2005). P-cadherin overexpression is an indicator of clinical outcome in invasive breast carcinomas and is associated with CDH3 promoter hypomethylation. Clin. Cancer Res..

[8] Stefansson I.M., Salvesen H.B., Akslen L.A. (2004). Prognostic impact of alterations in P-cadherin expression and related cell adhesion markers in endometrial cancer. J. Clin. Oncol..

[9] Zimmerman Z., Maniar T., Nagorsen D. (2015). Unleashing the clinical power of T cells: CD19/CD3 bi-specific T cell engager (BiTE®) antibody construct blinatumomab as a potential therapy. Int. Immunol..

[10] Lameris R., de Bruin R.C., Schneiders F.L., van Bergen en Henegouwen P.M., Verheul H.M., de Gruijl T.D., van der Vliet H.J. (2014). Bispecific antibody platforms for cancer immunotherapy. Crit. Rev. Oncol. Hematol..

[11] Carter P.J., Senter P.D. (2008). Antibody-drug conjugates for cancer therapy. Cancer J..

[12] Velders M.P., van Rhijn C.M., Oskam E.O., Fleuren G.J., Warnaar S.O., Litvinov S.V. (1998). The impact of antigen density and antibody affinity on antibody-dependent cellular cytotoxicity: Relevance for immunotherapy of carcinomas. Br. J. Cancer.

[13] Smith-Garvin J.E., Koretzky G.A., Jordan M.S. (2009). T cell activation. Annu. Rev. Immunol..

[14] Voskoboinik I., Whisstock J.C., Trapani J.A. (2015). Perforin and granzymes: Function, dysfunction and human pathology. Nat. Rev. Immunol..

[15] Baeuerle P.A., Reinhardt C. (2009). Bispecific T-cell engaging antibodies for cancer therapy. Cancer Res..

[16] Johnson S., Burke S., Huang L., Gorlatov S., Li H., Wang W., Zhang W., Tuaillon N., Rainey J., Barat B. (2010). Effector cell recruitment with novel fv-based dual-affinity re-targeting protein leads to potent tumor cytolysis and *in vivo* B-cell depletion. J. Mol. Biol..

[17] Moore P.A., Zhang W., Rainey G.J., Burke S., Li H., Huang L., Gorlatov S., Veri M.C., Aggarwal S., Yang Y. (2011). Application of dual affinity retargeting molecules to achieve optimal redirected T-cell killing of B-cell lymphoma. Blood.

[18] Ridgway J.B.B., Presta L.G., Carter P. (1996). “Knobs-into-holes” engineering of antibody CH3 domains for heavy chain heterodimerization. Protein Eng..

[19] Atwell S., Ridgway J.B.B., Wells J.A., Carter P. (1997). Stable heterodimers from remodeling the domain interface of a homodimer using a phage display library. J. Mol. Biol..

[20] Zhang C.C., Yan Z., Zhang Q., Kuszpit K., Zasadny K., Qiu M., Painter C.L., Wong A., Kraynov E., Arango M.E. (2010). PF-03732010: A fully human monoclonal antibody against P-cadherin with antitumor and antimetastatic activity. Clin. Cancer Res..

[21] Yoshioka H., Yamamoto S., Hanaoka H., Iida Y., Paudyal P., Higuchi T., Tominaga H., Oriuchi N., Nakagawa H., Shiba Y. (2012). *In vivo* therapeutic effect of CDH3/P-cadherin-targeting radioimmunotherapy. Cancer Immunol. Immunother..

[22] Suzuki S., Sano K., Tanihara H. (1991). Diversity of the cadherin family: Evidence for eight new cadherins in nervous tissue. Cell Regul..

[23] Fennell B.J., McDonnell B., Tam A.S., Chang L., Steven J., Broadbent I.D., Gao H., Kieras E., Alley J., Luxenberg D. (2013). CDR-restricted engineering of native human scFvs creates highly stable and soluble bifunctional antibodies for subcutaneous delivery. MAbs.

[24] Bluemel C., Hausmann S., Fluhr P., Sriskandarajah M., Stallcup W.B., Baeuerle P.A., Kufer P. (2010). Epitope distance to the target cell membrane and antigen size determine the potency of t cell-mediated lysis by bite antibodies specific for a large melanoma surface antigen. Cancer Immunol. Immunother..

[25] Perisic O., Webb P.A., Holliger P., Winter G., Williams R.L. (1994). Crystal structure of a diabody, a bivalent antibody fragment. Structure.

[26] Carmichael J.A., Power B.E., Garrett T.P.J., Yazaki P.J., Shively J.E., Raubischek A.A., Wu A.M., Hudson P.J. (2003). The crystal structure of an anti-CEA scFv diabody assembled from t84.66 scFvs in V_L_-to-V_H_ orientation: Implications for diabody flexibility. J. Mol. Biol..

[27] Roopenian D.C., Akilesh S. (2007). FcRn: The neonatal Fc receptor comes of age. Nat. Rev. Immunol..

[28] Kasaian M.T., Raible D., Marquette K., Cook T.A., Zhou S., Tan X.Y., Tchistiakova L. (2011). IL-13 antibodies influence IL-13 clearance in humans by modulating scavenger activity of IL-13Rα2. J. Immunol..

[29] Shapiro L., Weis W.I. (2009). Structure and biochemistry of cadherins and catenins. Cold Spring Harb. Perspect. Biol..

[30] Boggon T.J., Murray J., Chappuis-Flament S., Wong E., Gumbiner B.M., Shapiro L. (2002). C-cadherin ectodomain structure and implications for cell adhesion mechanisms. Science.

[31] Holliger P., Prospero T., Winter G. (1993). “Diabodies”: Small bivalent and bispecific antibody fragments. Proc. Natl. Acad. Sci. USA.

[32] Li L., Olafsen T., Anderson A., Wu A.M., Raubitschek A.A., Shively J.E. (2002). Reduction of kidney uptake in radiometal labeled peptide linkers conjugated to recombinant antibody fragments. Site-specific conjugation of DOTA-peptides to a cys-diabody. Bioconjug. Chem..

[33] Olafsen T., Cheung C., Yazaki P.J., Li L., Sundaresan G., Gambhir S.S., Sherman M.A., Williams L.E., Shively J.E., Raubitschek A.A. (2004). Covalent disulfide-linked anti-CEA diabody allows site-specific conjugation and radiolabeling for tumor targeting applications. Protein Eng. Des. Sel..

[34] Muller D., Karle A., Meissburger B., Hofig I., Stork R., Kontermann R.E. (2007). Improved pharmacokinetics of recombinant bispecific antibody molecules by fusion to human serum albumin. J. Biol. Chem..

[35] Stork R., Campigna E., Robert B., Muller D., Kontermann R.E. (2009). Biodistribution of a bispecific single-chain diabody and its half-life extended derivatives. J. Biol. Chem..

[36] Zugmaier G., Klinger M., Schmidt M., Subklewe M. (2015). Clinical overview of anti-CD19 BiTE^®^ and *ex vivo* data from anti-CD33 BiTE^®^ as examples for retargeting T cells in hematologic malignancies. Mol. Immunol..

[37] Carter P.J. (2011). Introduction to current and future protein therapeutics: A protein engineering perspective. Exp. Cell Res..

[38] Kranz D.M., Voss E.W. (1981). Partial elucidation of an anti-hapten repertoire in BALB/c mice: Comparative characterization of several monoclonal anti-fluorescyl antibodies. Mol. Immunol..

[39] Huang L., Johnson L.S. (2014). CD3-binding molecules capable of binding to human and non-human CD3.

[40] Chichili G.R., Huang L., Li H., Burke S., He L., Tang Q., Jin L., Gorlatov S., Ciccarone V., Chen F. (2015). A CD3×CD123 bispecific DART for redirecting host T cells to myelogenous leukemia: Preclinical activity and safety in nonhuman primates. Sci. Transl. Med..

[41] Finlay W.J., Bloom L., Cunningham O., Walls D., Loughran S.T. (2011). Optimized generation of high-affinity, high-specificity single-chain Fv antibodies from multiantigen immunized chickens. Protein Chromatography: Methods and Protocols.

[42] Wasley L.C., Rehemtulla A., Bristol A., Kaufman R.J. (1993). PACE/furin can process the vitamin K-dependent pro-factor IX precursor within the secretory pathway. J. Biol. Chem..

