# Supplementary material for: Development of PF-06671008, a Highly Potent Anti-P-cadherin/Anti-CD3 Bispecific DART Molecule with Extended Half-Life for the Treatment of Cancer
Source: Antibodies (Basel). 2016 Mar 4;5(1):6. doi: 10.3390/antib5010006 (PMC6698862; doi:10.3390/antib5010006)
Supplement: Supplementary file 1 [file antibodies-05-00006-s001.pdf]

Supplementary Materials: Development of PF-06671008, a Highly Potent Anti-P-cadherin/Anti-CD3 Bispecific DART Molecule with Extended Half-Life for the Treatment of Cancer

Adam R. Root [1,*], Wei Cao [1], Bilian Li [1], Peter LaPan [1], Caryl Meade [1], Jocelyn Sanford [1], Macy Jin [1], Cliona O'Sullivan [2], Emma Cummins [2], Matthew Lambert [2], Alfredo D. Sheehan [2], Weijun Ma [1], Scott Gatto [1], Kelvin Kerns [1], Khetemenee Lam[1], Aaron M. D'Antona [1], Lily Zhu [1], William A. Brady [1], Susan Benard [1], Amy King [1], Tao He [1], Lisa Racie [1], Maya Arai [1], Dianah Barrett [1], Wayne Stochaj [1], Edward R. LaVallie [1], James R. Apgar [1], Kristine Svenson [1], Lidia Mosyak [1], Yinhua Yang [3], Gurunadh R. Chichili [3], Liqin Liu [3], Hua Li [3], Steve Burke [3], Syd Johnson [3], Ralph Alderson [3], William J. J. Finlay [2], Laura Lin [1], Stéphane Olland [1], William Somers [1], Ezio Bonvini [3], Hans-Peter Gerber [4], Chad May [4], Paul A. Moore [3], Lioudmila Tchistiakova [1] and Laird Bloom [1]

S.1. Generation of Recombinant P-Cadherin Protein Containing Human IgG F, His6 or Avi/FLAG Tags

Recombinant P-cadherin extracellular domain (ECD) proteins were either purchased commercially (R & D Systems number 861-PC/761-MP, Minneapolis, MN, USA) or generated in-house at Pfizer. For constructs generated in-house, complementary deoxyribonucleic acid (cDNA) was obtained for human and mouse P-cadherin ECD (accession number NM_001793.3; NM_007665.3) from a commercial source (Origene, Rockville, MD, USA). Using conventional molecular biological techniques or through gene synthesis at an external vendor (Blue Heron, Bothell, WA, USA), the ECD fragment (including pro-peptide region) was cloned into a Pfizer-proprietary mammalian expression vector containing a mouse IgG leader sequence, the canonical Factor Xa cleavage sequence IEGRMD, human IgG1 Fc domain and 6-HIS tag. cDNA constructs encoding the human or murine P-cadherin ECD with a signal peptide at N-terminus and an Avi and a FLAG tag at the C-terminus were also synthesized and cloned into a Pfizer-proprietary mammalian expression vector. An expression vector containing the cynomolgus monkey P-cadherin ECD cDNA genetically linked to a His_6 tag was obtained from colleagues in Pfizer. All vectors were sequence-confirmed and transiently transfected into FreeStyle™ 293 human embryonic kidney cells (HEK) (Life Technologies, Grand Island, NY, USA) according to the manufacturer's method and expressed over 5–7 days. Pro-peptide processing of P-cadherin was determined to be about 50% or less in these samples. For enhanced processing of the pro-peptide, an expression vector containing the paired basic amino acid cleaving enzyme (PACE) was co-transfected along with the P cadherin constructs [42]. For human IgG Fc-containing constructs, soluble protein of interest was purified using standard protein A chromatographic techniques (Protein A FF, GE Healthcare, Piscataway, NJ, USA) followed by gel filtration size exclusion chromatography (Superdex200, GE Healthcare, Piscataway, NJ, USA). For Avi/FLAG-containing constructs, conditioned medium was batch bound to 10 mL FLAG M2 resin (Sigma-Aldrich, St. Louis, MO, USA) for 2 h at 4 °C. Resin was washed with 5 column volumes (CV) of phosphate buffered saline (PBS) then eluted with 5 CV of PBS + 100 mM FLAG peptide. For some constructs, the resin was further eluted with 100 mM Glycine pH 3 and neutralized in 10% final volume 1 M Tris pH 8.5. Fractions containing hu P-Cadherin were pooled and concentrated on a 30 kD MWCO Vivaspin 3 kD concentrator (Sartorius, Bohemia, NY, USA). Concentrated protein was then dialyzed into PBS + Ca^{2+} and Mg^{2+}. Cynomolgus monkey P-cadherin ECD-His_6 was was purified as follows: Conditioned medium was batch bound to 10 mL Ni NTA fast flow resin (Qiagen, Valencia, CA) for 2 h at 4 °C. Protein was washed with 7 CV of PBS and then eluted, first with 7 CV PBS + 10 mM imidazole, with then 7 CV of PBS + 20 mM imidazole, and finally with 7 CV of PBS + 100 mM imidazole. Fractions from the 20 and 200 mM imidazole elution steps containing cynomolgus monkey P-cadherin were pooled and concentrated on a Vivaspin 3K cutoff concentrator (Sartorius, Bohemia, NY, USA). Concentrated protein was then dialyzed into PBS + Ca^{2+} and Mg^{2+}. Purified protein was characterized for purity and activity by binding ELISA using

commercially-available anti-human P-cadherin monoclonal and polyclonal antibodies (R & D Systems, Minneapolis, MN, USA). Purified recombinant human CD3 epsilon-delta protein was acquired from MacroGenics.

S.2. Development of Engineered Cell Lines Expressing P-Cadherin

P-cadherin ortholog expressing cell lines were generated by performing stable transfections in Chinese Hamster Ovary (CHO-Dukx) or SW480 primary colon (ATCC CCL-228) cell lines. cDNA was obtained for human (catalog number SC303069; accession number NM_001793.3) and mouse P-cadherin ECD (catalog number MC221848; accession number NM_007665.3) from a commercial source (Origene, Rockville, MD, USA). Using conventional molecular biological techniques, the full gene (including pro-peptide region) was cloned into a Pfizer-proprietary mammalian expression vector sequence-confirmed. Pilot expression analysis indicated that the pro-peptide processing of P-cadherin ECD was about 50% or less. For enhanced pro-peptide processing, CHO cells harboring soluble PACE stably integrated in its genome were used [42]. Following transfection of the expression vector encoding the P-cadherin ortholog with the Lipofectamine LTX and Plus™ reagent (Life Technologies, Grand Island, NY, USA), CHO cells were selected in 20, 50, 100 nM Methotrexate (MTX) + 1 mg/mL G418 (Geneticin, Life Technologies, Grand Island, NY, USA) with 20 nM MTX chosen for lead human clonal line and 100 nM for lead murine clonal line.

S.3. Preparation of Phage Expressing scFv for Use in ELISAs

To prepare phage expressing scFv on their surface, 96-deep well plates containing 1 mL 2× YT media with 2% glucose/100 μg/mL ampicillin were inoculated with 0.5–1 μL from thawed glycerol stocks (one clone per well) using the QPix™ 2 Colony picker (Molecular Devices, Sunnyvale, CA, USA) and grown at 37 °C (900 rpm) for ~4 h. Next, 5 μL of a 1:29 dilution of helper phage (8.3×10^{13} pfu) was added and the plates and incubated for a further 30 min at 37 °C with no shaking then 1 h at 300 rpm. Plates were centrifuged and the media was replaced with a kanamycin/non-glucose containing media (2× YT with 50 μg/mL kanamycin and 100 μg/mL ampicillin). Plates were grown overnight at 25 °C (900 rpm), and phage were harvested in the supernatant following centrifugation.

S.3.1. Transient DART Protein Expression

For transient transfections, expression vectors encoding the DART protein variants were co-transfected into HEK FreeStyle™ 293 cells as previously described (Johnson *et al.*, 2010). Supernatants were collected on Day 6 and screened by ELISA for binding to P-cadherin recombinant protein and expressed on the cell surface. Expression yields were determined by Octet concentration assay (Pall, Port Washington, NY, USA). Protein A-labeled biosensors dipped in conditioned medium supernatants or controls using the Octet QK, according to the manufacturer's instructions, and DART protein concentrations were calculated using a pre-determined standard curve.

S.3.2. Purification of E-K DART Protein

DART proteins retaining P-cadherin affinity were selected for purification. DART proteins were affinity purified using an anti-coiled-coil mAb 15F1 coupled to CNBr-activiated sepharose 4B (GE Healthcare, Piscataway, NJ, USA). The affinity Sepharose resin was equilibrated in PBS, pH 7.2 before loading. Following loading, bound protein was washed with 5 column volumes (CV) equilibration buffer and eluted with 2 CV 50 mM glycine, pH 2.5 then immediately neutralized with 1 M Tris-HCl pH 8.5. The DART proteins were further purified by size exclusion chromatography (SEC) using a Superdex200HR 10/30 according to the manufacturer's protocol (GE Healthcare, Piscataway, NJ, USA). Purified DART proteins were analyzed by SDS-PAGE and analytical SEC as previously described [16].

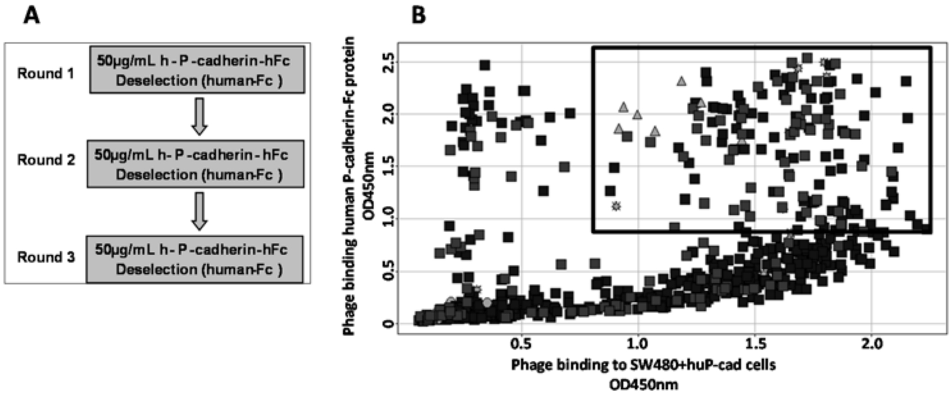

Figure S1. (**A**) Schematic representation of the phage selection strategy using recombinant protein for naïve library selections. (**B**) Phage display screening enzyme-linked immunosorbent assays (ELISAs) against recombinant human P-cadherin-Fc protein and SW480 cells overexpressing human P-cadherin. Clones that demonstrated strong binding activity to both recombinant protein (OD450 nm > 1) and cell surface-expressed P-cadherin (OD450 nm > 0.8) were selected (boxed area). Light and dark squares represent scFv-expressing phage; Triangle = positive control antibody; Star = positive control scFv-expressing phage; Circle = negative control scFv-expressing phage.

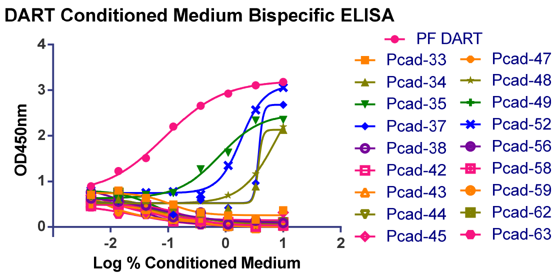

Figure S2. Bispecific binding of anti-P-cadherin dual affinity re-targeting (DART) proteins in conditioned medium against human CD3 and human P-cadherin-Fc. DARTs exhibiting simultaneous binding activity were selected for tumor cell lysis activity in cytotoxic T-lymphocyte (CTL) assays.

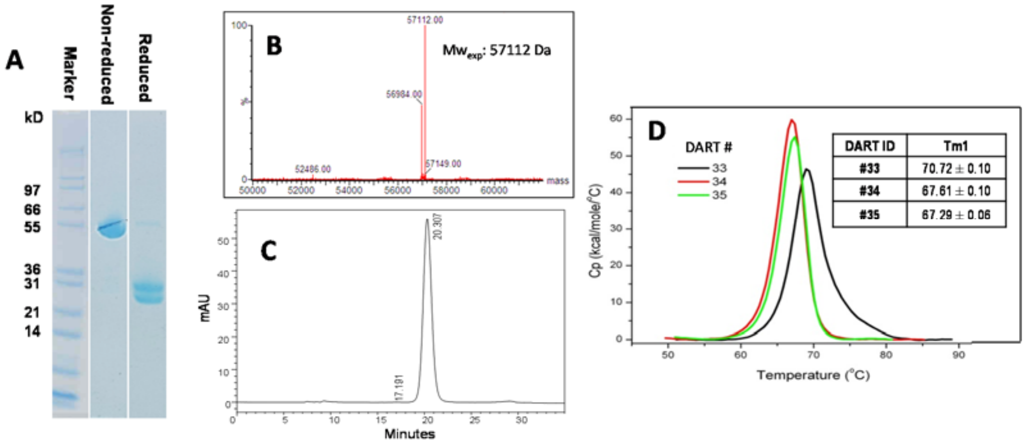

Figure S3. (**A**) SDS-PAGE analysis of purified anti-P-cadherin DART 35 under non-reducing and reducing conditions. (**B**) Non-reducing liquid chromatography/ mass spectrometry (LC/MS) analysis of DART 35. The peak at 57112 Da corresponds to the correctly paired heterodimer of DART 35; the 56984 Da peak is assigned to the loss of Lysine amino acid from one of the sequences. LC/MS analysis demonstrated that the majority of detected DART molecular weight matches the predicted heterodimer sequence. (**C**) Size exclusion chromatography (SEC) of DART 35 on Superdex200 10/30GL column. (**D**) Differential scanning calorimetry (DSC) analysis of phage derived DARTs exhibit thermal profiles with Tm1 transitions ≥ 67 °C.

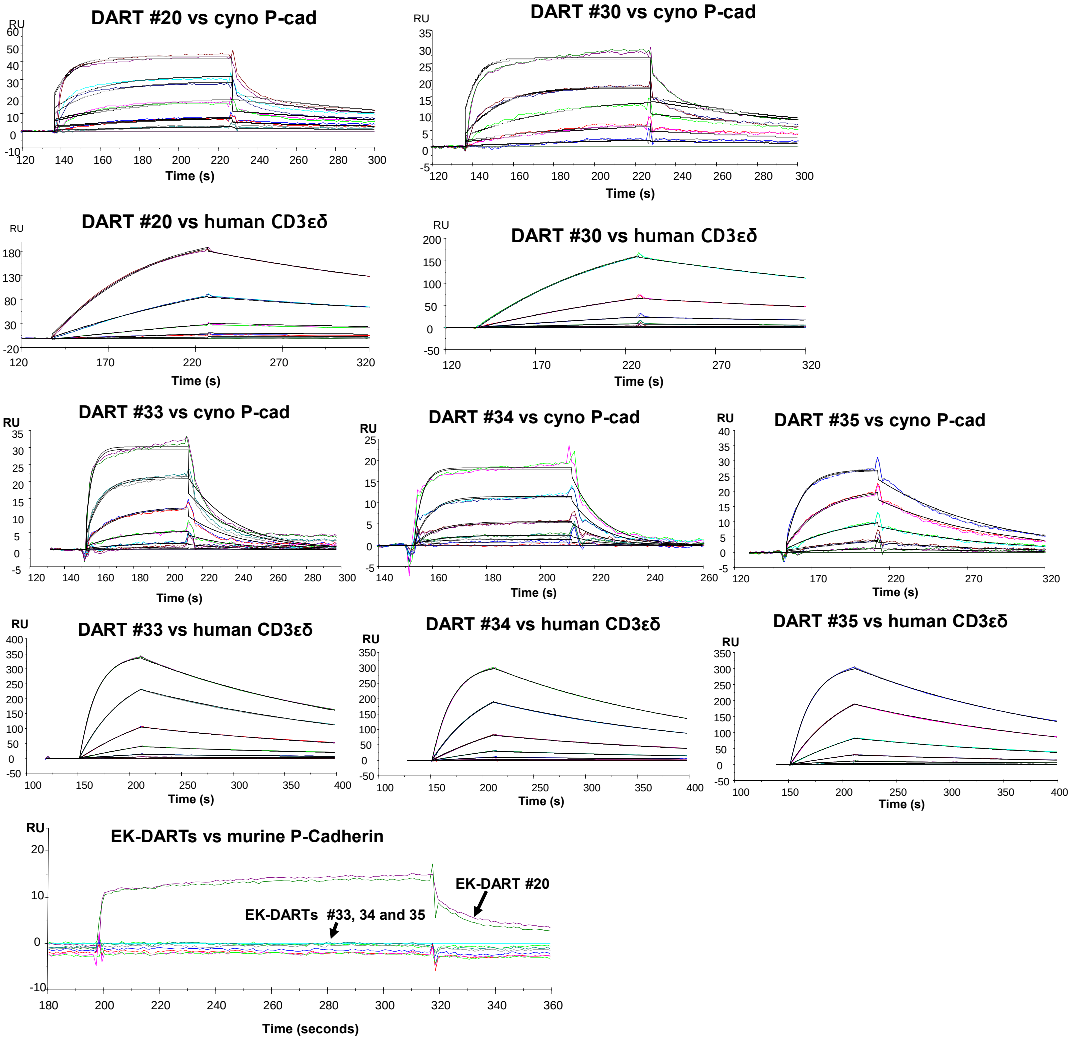

Figure S4. Kinetic surface plasmon resonance (SPR) analysis of anti-P-cadherin DARTs 20, 30, 33, 34 and 35 binding to cynomolgus P-cadherin, human CD3, and murine P-cadherin captured or immobilized on the surface. Colored lines represent the fit to a 1:1 Langmuir model of the experimental binding curves obtained at DART concentrations of 1.1, 3.3, 10, 30, 90 or 270 nM. DARTs 33, 34 and 35 do not bind to murine P-cadherin.

A

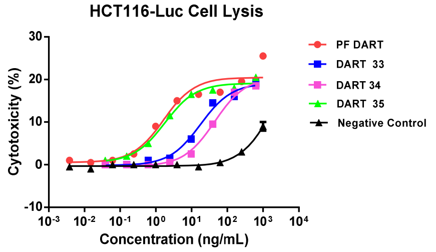

B

DART ID	CHO-CDh3 (24h) EC_{50} (nM)	H1650 (24h) EC_{50} (nM)	HCT-116-Luc (24h) EC_{50} (nM)
DART 33	0.309	1.246	1.03
DART 34	0.7365	1.888	3.83
DART 35	0.0398	0.1042	0.10
PF DART	0.0284	0.0463	0.06
DART 20	NR*	ND	NT
DART 30	NR*	NR*	NT

Figure S5. (**A**) CTL directed killing of NCI-H1650 adenocarcinoma cells by P-cadherin DARTs. (**B**) Summary table of tumor and Chinese hamster ovary (CHO) cell lysis by phage derived DART proteins. * = samples were tested on a separate date with different donor effector cells. ND = not determined; NR = no response; NT = not tested.

Table S1. SPR equilibrium dissociation constants for the binding of DART proteins against (**A**) cynomolgus P-cadherin extracellular domain (ECD) and (**B**) human soluble CD3. (**C**) Binding summary of top phage derived anti-P-cadherin DARTs against P-cadherin, E-cadherin, VE-cadherin, NCI-H1650 primary lung adenocarcinoma cells and parental CHO cells. The top phage derived DARTs exhibited specific P-cadherin binding activity to recombinant protein and cell-surface expressed P-cadherin.

A

Equilibrium dissociation constants (K_D) for the binding of anti-P-cadherin EK-DARTs to cynomolgus monkey P-cadherin

DART ID	Association rate constant (k_a) ($M^{-1}\cdot s^{-1}$)	Dissociation rate constant k_d (s^{-1})	Equilibrium dissociation constant (K_D) (±SD) (nM)
DART 33	1.15E+06	5.78E−02	46.4 ± 4.1
DART 34	7.05E+05	8.33E−02	129.25 ± 31.75
DART 35	3.18E+05	1.38E−02	43.4 ± 0.1
PF DART	2.08E+06	4.49E−03	2.12 ± 0.71
DART 20	3.46E+06	7.04E−03	2.03 ± 0.07
DART 30	4.72E+06	6.48E−03	1.37 ± 0.11

B

Equilibrium dissociation constants (K_D) for the binding of anti-P-cadherin EK-DARTs to Human CD3 epsilon

DART ID	Association rate constant (k_a) ($M^{-1}\cdot s^{-1}$)	Dissociation rate constant k_d (s^{-1})	Equilibrium dissociation constant (K_D) (±SD) (nM)
DART 33	2.28E+05	4.03E−03	18.25 ± 0.55
DART 34	1.95E+05	4.45E−03	21.85 ± 0.95
DART 35	1.89E+05	4.47E−03	23.15 ± 0.45
PF DART	6.59E+05	3.78E−03	6.80 ± 2.81
DART 20	9.79E+05	6.09E−03	5.19 ± 0.35
DART 30	3.53E+05	4.57E−03	13.6 ± 3.49

C

DART ID	Hu P-cadherin-Fc EC_{50} (nM)	E-cadherin-Fc EC_{50} (nM)	VE-cadherin-Fc EC_{50} (nM)	H1650 EC_{50} (nM)	CHO Parental EC_{50} (nM)
DART 33	1.8	NB	NB	60.1	NB
DART 34	2.85	NB	NB	67.95	NB
DART 35	1.92	NB	NB	24.87	NB
PF DART	2.1	NB	NB	19.69	NB
DART 20	12.3	NB	NB	~114.7	NB
DART 30	3.68	NB	NB	141.9	NB
Neg Control DART	NB	NB	NB	NB	NB

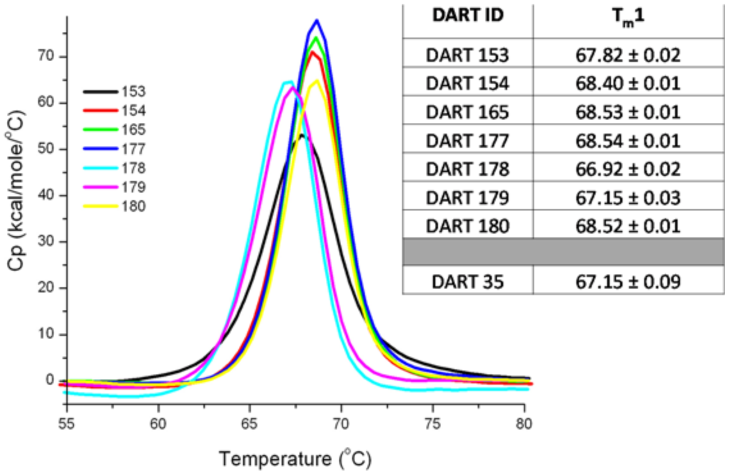

Figure S6. Differential scanning calorimetry (DSC) thermal stability analysis of affinity optimized DARTs show similar melting transitions to the parental DART 35.

Table S2. SPR equilibrium dissociation constants for the binding of affinity matured DART proteins against (**A**) cynomolgus P-cadherin ECD and (**B**) human soluble CD3.

A

Equilibrium dissociation constants (K_D) for the binding of affinity matured anti-P-cadherin EK-DARTs to cynomolgus monkey P-cadherin determined by SPR (Biacore)

DART ID	Association rate constant (k_a) ($M^{-1}\cdot s^{-1}$)	Dissociation rate constant k_d (s^{-1})	Equilibrium dissociation constant (K_D) (±SD) (pM)	Fold increase in K_D over parental DART #35
DART 35	3.18E+05	1.38E−02	43400 ± 100	-
DART 153	7.32E+05	1.72E−05	231.5 ± 17.5	187.5
DART 154	1.04E+06	3.90E−03	3930.0 ± 910	11.0
DART 165	8.34E+05	3.09E−04	370.0 ± 87	117.3
DART 177	8.02E+05	3.83E−04	483.0 ± 82	89.9
DART 178	1.67E+05	2.16E−04	1310.0 ± 140	33.1
DART 179	1.11E+06	4.58E−03	4165.0 ± 255	10.4
DART 180	7.71E+05	7.44E−04	965.5 ± 19.5	45.0

B

Equilibrium dissociation constants (K_D) for the binding of affinity matured anti-P-cadherin EK-DARTs to Human CD3 epsilon determined by SPR (Biacore)

DART ID	Association rate constant (k_a) ($M^{-1}\cdot s^{-1}$)	Dissociation rate constant k_d (s^{-1})	Equilibrium dissociation constant K_D) (±SD) (nM)	Fold increase in K_D over parental DART #35
DART 35	1.89E+05	4.47E−03	23.15 ± 0.45	-
DART 153	4.03E+05	6.31E−03	15.65 ± 1.75	1.5
DART 154	4.56E+05	7.68E−03	17.10 ± 0.90	1.4
DART 165	2.78E+05	4.17E−03	14.90 ± 2.80	1.6
DART 177	2.53E+05	4.43E−03	19.95 ± 6.55	1.2
DART 178	2.30E+05	5.26E−03	23.10 ± 1.10	1.0
DART 179	3.06E+05	6.41E−03	25.75 ± 9.25	0.9
DART 180	7.06E+05	1.11E−02	15.9 ± 1.00	1.5

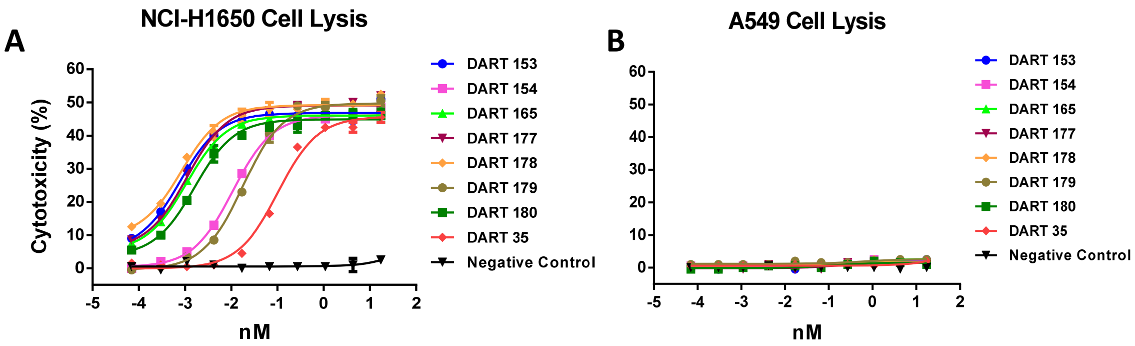

C

DART ID	HCT116-Luc 48hr 10:1 EC_{50} (nM)	H1650 LDH 24hr 30:1 EC_{50} (nM)	A549 LDH 24hr 30:1 EC_{50} (nM)
DART 153	0.0005	0.0008	NR
DART 154	0.0166	0.0107	NR
DART 165	0.0015	0.0011	NR
DART 177	0.0007	0.0011	NR
DART 178	0.0018	0.0008	NR
DART 179	0.0432	0.0178	NR
DART 180	0.0018	0.0016	NR
DART 280	NT	0.0012*	NR*
DART 35	0.2051	0.1016	NR

Figure S7. Graphical representation of CTL assay of affinity optimized DARTs against (**A**) NCI-H1650 or (**B**) A549 adenocarcinoma cells co-incubated with peripheral blood mononuclear cells (PMBCs) over 24 h. (**C**) Optimized DARTs were evaluated for cytotoxicity against HCT-116 colorectal line (co-transfected with luciferase) and NCI-H1650 adenocarcinoma cell line using human PBMCs. * = samples were tested on a separate date with different donor effector cells.

A

Protein A Recovery	OD 280	ext.	conc (mg/mL)	Recovery (mg)	Yield (mg/L)
HUPCADECD1	0.89	1.26	0.71	2.77	138.31
HUPCADECD1-2	0.67	1.08	0.62	2.43	121.27
HUPCADECD1-3	1.09	1.02	1.07	4.18	209.00
HUPCADECD1-4	1.16	1.00	1.16	4.52	225.95
HUPCADECD1-5	0.48	1.02	0.47	1.82	90.93

Final Recovery	OD 280	ext.	conc (mg/mL)	Recovery (mg)	Yield (mg/L)
HUPCADECD1	0.81	1.26	0.64	2.37	118.68
HUPCADECD1-2	0.61	1.08	0.57	2.11	105.43
HUPCADECD1-3	1.01	1.02	0.99	3.66	182.88
HUPCADECD1-4	1.10	1.00	1.10	4.06	203.25
HUPCADECD1-5	0.46	1.02	0.45	1.66	83.25

B

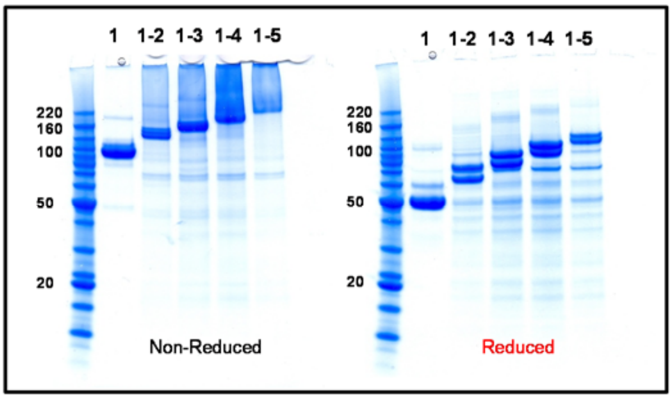

C

Sample	Length	MW (kDa)
HUPCADECD1	355	39.6
HUPCADECD1-2	468	51.8
HUPCADECD1-3	582	64.4
HUPCADECD1-4	688	75.8
HUPCADECD1-5	795	87.8

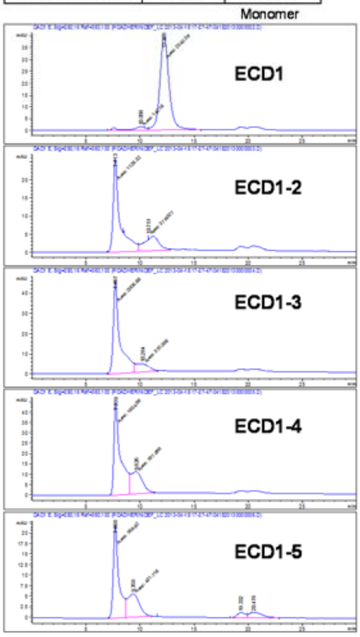

Figure S8. (**A**) P-cadherin ECD truncated constructs were expressed transient transfection in human embryonic kidney (HEK) 293 cells in 20 mL over 5 days and purified by protein A and buffer exchange into phosphate-buffered saline (PBS). Expression yields ranged from 80–200 mg/L; (**B**) SDS-PAGE analysis of truncated ECD-Fc constructs shows bands at expected molecular weight (MW) in reduced and non-reduced gels. A second band in the reduced gel suggests poor cleavage of the pro-peptide; (**C**) Analytical SEC shows high levels of high molecular mass species.

Table S3. Data collection and refinement statistics.

**Data Collection**	Value
Space group	P 3 21
Unit cell (a,b,c) (Å)	142.81, 142.81, 62.69
Wavelength (Å)	1
Resolution range (Å)	123.7–2.5 (2.12–2.01)
Total reflections	59,092
Unique reflections	6436
Completeness (%)	91
Redundancy	9.2
I/s(I)	12.4 (2.4)
R_{merge} [a] (%)	14.2 (82.4)
Refinement	
Resolution range (Å)	29.4–2.01 (2.06–2.01)
R_{work} (%)	17.6 (21.6)
R_{free} (%)	20.5 (25.2)
rmsd bond length (Å)	0.01
rmsd bond angle (°)	1.08
Average B-factor (Å^2)	32.4
Protein Atoms	3529
Solvent Atoms	574
Heterogen Atoms	599

$R_{merge} = |I_h - \langle I_h \rangle|/I_h$, where $\langle I_h \rangle$ is the average intensity over symmetry equivalents. I/s(I) = averageI/averages(I); $R_{work} = ||F_{obs}| - |F_{calc}||/|F_{obs}|$; $R_{free\ (\%)}$ is equivalent to R_{work}, but calculated for a randomly chosen 5% of reflections omitted from the refinement process. Values in parentheses represent the corresponding values for the highest resolution shells.

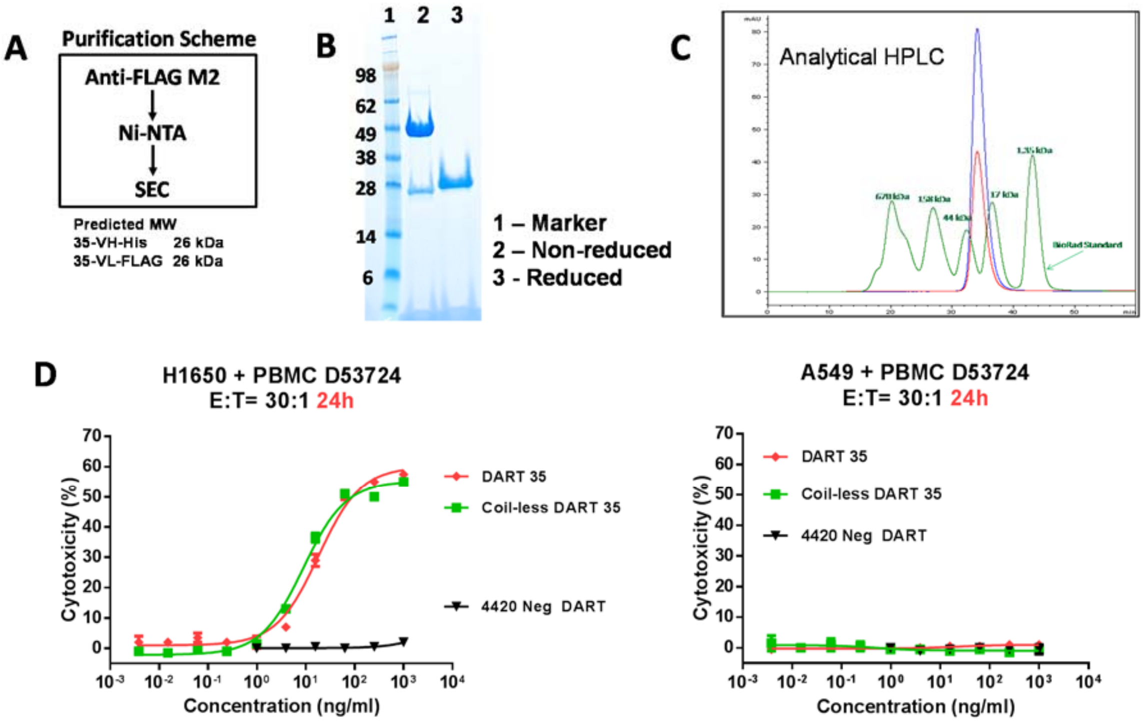

Figure S9. (**A**) The coil-less DART 35 was purified first with anti-FLAG M2 capture step followed by a Ni-NTA (Qiagen) second step for removal of homodimer, Purified heterodimer was then loaded onto a Superdex200 size exclusion column for removal of high molecular weight (HMW) species. (**B**) SDS-PAGE analysis of the final SEC pool showed pure bands at the expected molecular weights; (**C**) Analytical SEC analysis showed high purity of the heterodimeric DART protein (Blue = 280 nm; Red = 260 nm; Green = MW Standard (BioRad); (**D**) NCI-H1650 (P-cad+) and A549 (P-cad−) adenocarcinoma cell lysis by DART 35 and coil-less DART 35 (used for crystallography) using human PBMCs. Coil-less DART 35 shows CTL activity equivalent to that of DART 35.

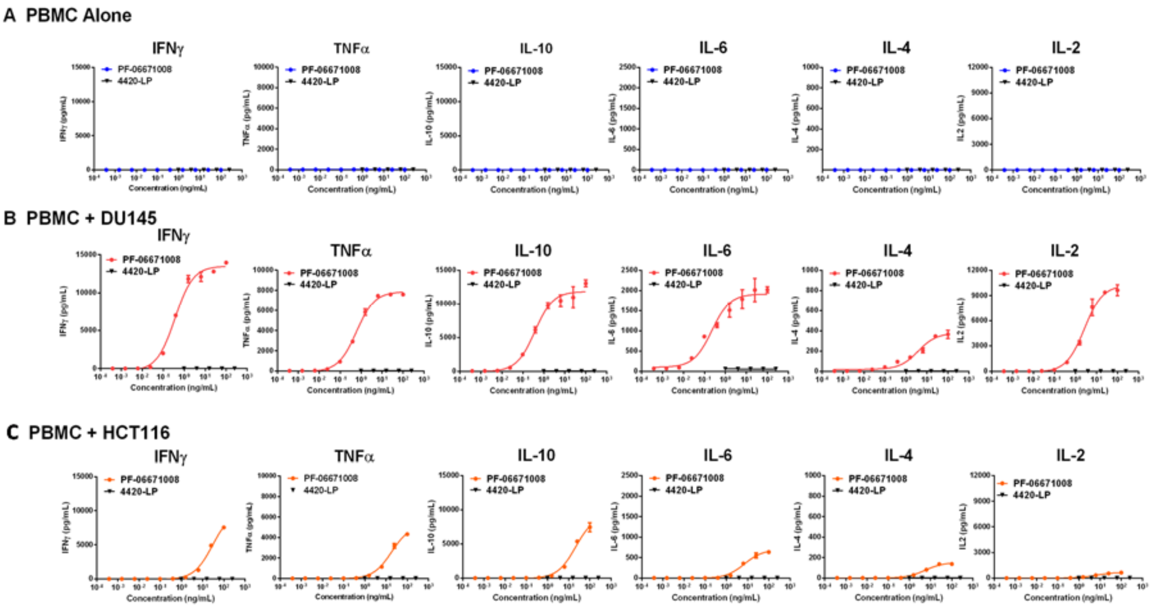

Figure S10. Cytokine release from human PBMCs in the presence or absence of target cells following treatment with PF-06671008. Human PBMCs were treated with PF-06671008 or control DART protein (4420-LP) for approximately 24 h alone (**A**) or in the presence of DU145 or HCT116-Luc target cells (**B**,**C**). Culture supernatants were collected and subjected to ELISA-based cytokine measurement. Y-axes are the concentrations of cytokine released (pg/mL) and x-axes are the concentrations of PF-06671008 or 4420-LP (ng/mL).

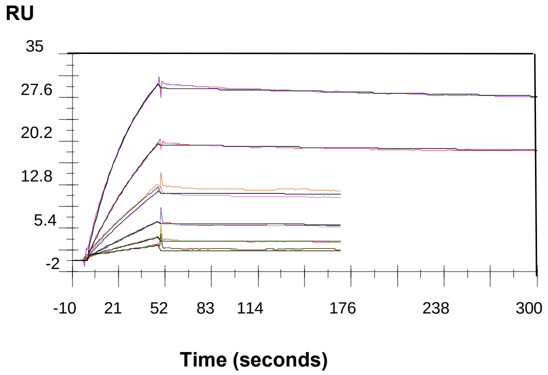

Figure S11. Kinetic SPR analysis of anti-P-cadherin PF-06671008 to cynomolgus P-cadherin immobilized on the surface. Colored lines represent the fit to a 1:1 Langmuir model of the experimental binding curves obtained at DART concentrations of 3.13, 6.25, 12.5, 25, 50 or 100 nM.

© 2016 by the authors; licensee MDPI, Basel, Switzerland. This article is an open access article distributed under the terms and conditions of the Creative Commons by Attribution (CC-BY) license (http://creativecommons.org/licenses/by/4.0/).
